# Defect Engineering of Disordered Carbon Anodes with Ultra-High Heteroatom Doping Through a Supermolecule-Mediated Strategy for Potassium-Ion Hybrid Capacitors

**DOI:** 10.1007/s40820-022-01006-0

**Published:** 2023-01-27

**Authors:** Lei Zhao, Shirong Sun, Jinxin Lin, Lei Zhong, Liheng Chen, Jing Guo, Jian Yin, Husam N. Alshareef, Xueqing Qiu, Wenli Zhang

**Affiliations:** 1 Guangdong Provincial Key Laboratory of Plant Resources Biorefinery, School of Chemical Engineering and Light Industry, Guangdong University of Technology (GDUT), 100 Waihuan Xi Road, Panyu District, Guangzhou, 510006 People’s Republic of China; 2 Jieyang Branch of Chemistry and Chemical Engineering Guangdong Laboratory (Rongjiang Laboratory), Jieyang, 515200 People’s Republic of China; 3 Key Laboratory of Dielectric and Electrolyte Functional Material Hebei Province, School of Resources and Materials, Northeastern University at Qinhuangdao, Qinhuangdao, 066004 People’s Republic of China; 4 Materials Science and Engineering, Physical Science and Engineering Division, King Abdullah University of Science and Technology (KAUST), Thuwal, 23955-6900 Saudi Arabia; 5 School of Advanced Manufacturing, Research Institute of Green Chemical Engineering and Advanced Materials, Guangdong University of Technology (GDUT), Jieyang, Jieyang, 515200 People’s Republic of China

**Keywords:** Defect, Heteroatom, Active sites, Supramolecule, Potassium-ion hybrid capacitors

## Abstract

Amorphous carbons are promising anodes for high-rate potassium-ion batteries. Most low-temperature annealed amorphous carbons display unsatisfactory capacities. Heteroatom-induced defect engineering of amorphous carbons could enhance their reversible capacities. Nevertheless, most lignocellulose biomasses lack heteroatoms, making it a challenge to design highly heteroatom-doped carbons (> 10 at%). Herein, we report a new preparation strategy for amorphous carbon anodes. Nitrogen/sulfur co-doped lignin-derived porous carbons (NSLPC) with ultra-high nitrogen doping levels (21.6 at% of N and 0.8 at% of S) from renewable lignin biomacromolecule precursors were prepared through a supramolecule-mediated pyrolysis strategy. This supermolecule/lignin composite decomposes forming a covalently bonded graphitic carbon/amorphous carbon intermediate product, which induces the formation of high heteroatom doping in the obtained NSLPC. This unique pyrolysis chemistry and high heteroatom doping of NSLPC enable abundant defective active sites for the adsorption of K^+^ and improved kinetics. The NSLPC anode delivered a high reversible capacity of 419 mAh g^‒1^ and superior cycling stability (capacity retention of 96.6% at 1 A g^‒1^ for 1000 cycles). Potassium-ion hybrid capacitors assembled by NSLPC anode exhibited excellent cycling stability (91% capacity retention for 2000 cycles) and a high energy density of 71 Wh kg^–1^ at a power density of 92 W kg^–1^.

**Supplementary Information:**

The online version contains supplementary material available at 10.1007/s40820-022-01006-0.

## Highlights

- The N/S co-doped lignin-derived porous carbon (NSLPCs) with ultra-high heteroatom doping was prepared through a novel supramolecule-mediated pyrolysis strategy.
- Covalently bonded graphitic carbon/amorphous carbon intermediates induce the formation of high heteroatom doping.
- The high heteroatom doping of NSLPC could provide abundant defective active sites for the adsorption of K^+^.

**Supplementary Information:**

The online version contains supplementary material available at 10.1007/s40820-022-01006-0.

## Graphical abstract

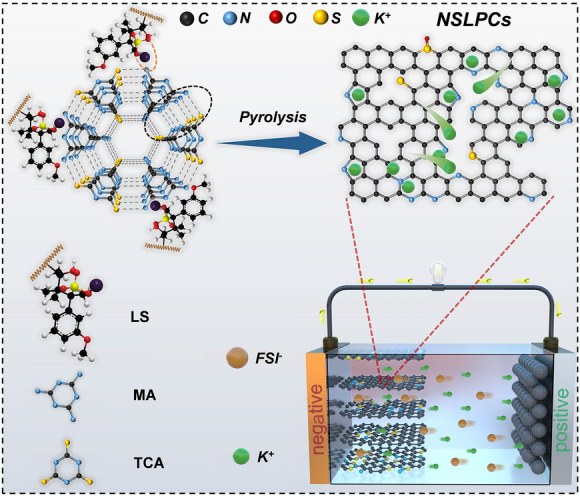

## Introduction

Lithium-ion batteries (LIBs) have been playing an indispensable role in hand-held electronics, electric vehicles, and electrical energy storage since their invention in 1991 [1–3]. However, considering much more abundant resources of potassium (1.5 wt%) than lithium (0.0017 wt%) in the earth crust [4, 5], potassium-ion batteries (PIB) family ushers in a more promising future in the field of next-generation electrochemical energy storage [6]. Potassium-ion hybrid capacitors (PIHCs) [7], in particular, combine the advantages of high power density of capacitors and high energy density of rechargeable batteries [8, 9]. Benefiting from the merits of high conductivity, low cost, and abundant precursors, carbon is inescapably regarded as one of the most promising anode materials for the preparation of PIHC [10, 11]. However, commercial graphite anode exhibits moderate electrochemical performance (278 mAh g^–1^) [12, 13], inferior rate capability, and poor cycling stability due to significant volume expansion variation (61%) and low diffusion coefficient of K^+^ [14–16]. Therefore, high-rate carbon anodes with rapid K^+^ storage capabilities and high capacities need to be rationally designed for PIHC.

Low-temperature pyrolyzed amorphous carbons with large interlayer spacing (> 3.7 Å) could provide excellent rate performance for the anodes of PIHCs due to their tunable structure (large layer spacing, abundant defects, and rich pores) and abundant adsorption active sites [4]. The excellent potassium storage performance of amorphous carbon is mainly ascribed to the adsorption/desorption of K^+^ in the defect sites [17, 18], which is different from the (de)intercalation mechanism of graphite [19–21]. However, most amorphous carbons show low capacities because of the presence of cross-linked sp^3^ linkage in carbon skeletons. Heteroatom doping is commonly used to break these cross-links and induce heteroatom-induced or carbon-vacancy defects [22]. Therefore, in order to enhance the capacity and rate performance of amorphous carbon anodes, it is of great importance to increase their edge defect sites and layer spacing. Hard carbons treated at high temperature (> 1000 °C) display limited capacities (< 300 mAh g^‒1^) and inferior rate capabilities due to their relatively small interlayer spacing, cross-linked graphene lattices and low defect density [23]. Meanwhile, hard carbons show poor potassium storage performances due to the intercalation mechanism of K^+^ stored in the low potential range. Therefore, low-temperature pyrolyzed amorphous carbons with mainly adsorption mechanism are usually investigated as the anodes for PIHCs. Heteroatom doping (N, S, O, B, etc*.*) [24, 25] could induce abundant defect sites in the amorphous carbon skeleton to enhance their capacities and rate capability [17, 26].

Primary biomass (rice husk, wood, vegetable, etc*.*) [3, 27] and its components (lignin [28, 29], cellulose [30], etc.) have been extensively studied as the precursors for preparing amorphous carbons anodes. However, amorphous carbon anodes prepared with primary biomass or its constituents have low nitrogen doping levels (< 10 at%), which results in the inability to achieve high capacities and high-rate capabilities [31–34].

The commonly used heteroatom doping strategies include the carbonization of biomass or biomolecules through chemical grafting, direct mixing, and covalent bonding with doping agents [35, 36]. Yang et al. [37] prepared SiO_2_@lignin amine urea–formaldehyde resin with 6.03 at% nitrogen doping level by chemically grafting lignin with urea. Yang et al. [38] fabricated three-dimensional porous carbon with 5.73 at% nitrogen doping by mixing bagasse and urea. The intrinsic reason for low nitrogen doping levels in the obtained amorphous carbons is the difficulty in bonding heteroatoms at the molecular level to carbon skeleton during pyrolysis.

Based on the discussion above, we propose a supermolecule-mediated method to prepare ultra-high N/S-doped lignin-derived porous carbon materials by homogeneously mixing biomolecule with nitrogen doping agents at the molecular level to achieve high heteroatom doping level and excellent potassium storage performance. Lignin, one of the abundant renewable biomolecules, was selected as an example to demonstrate our general strategy for preparing highly N-doped carbon anodes using general biomasses. The nitrogen/sulfur-containing supermolecule could form a molecule-level dispersed state with lignin, which induces the high doping efficiency of heteroatoms. The supermolecule could form a C_3_N_4_ intermediate covalently bonded with carbon skeleton formed from lignin. Notably, the obtained carbon shows an ultra-high N atom doping level (21.6 wt%) and a high edge nitrogen doping level (18.3 at% of pyridinic-N and pyrrolic-N dopants). Meanwhile, a large number of defects in NSLPCs are formed, which is attributed to the rapid liberation of intermediate gases (CS_2_, NH_3_, CO, CO_2_) and the decomposition of nitrogen-containing molecular fragments. The unique defect-rich structure with abundant edge defects adsorbs large amounts of K^+^, thus improving potassium-ion storage performance. We provide a general synthesis methodology to achieve high heteroatom doping in amorphous carbons using general biomass constitutes.

## Experimental Methods

### Preparation of NSLPCs

At room temperature, 0.1 mol melamine (MA) was put into 100 mL of deionized water and stirred vigorously, which was noted as solution A. 2 g sodium lignosulfonate (LS) was introduced into solution A and stirred until completely dissolved, which was noted as solution B. Then 0.1 mol trithiocyanuric acid (TCA) was added to the mixed solution B and stirred for 12 h, which was noted as solution C. Finally, solution C was transferred to a hydrothermal reactor and reacted at 100 °C for 12 h to obtain the precursor. The N/S co-doped lignin-derived porous carbons (NSLPCs) were obtained by calcination of the prepared precursors at temperatures of 700–1000 °C (protocol: room temperature to 550 °C hold for 1 h and the temperature was increased to 700–1000 °C hold for 2 h) in N_2_ atmosphere with a flow rate of 60 sccm under a heat-ramping rate of 5 °C min^−1^. For comparison, LS was calcined in a nitrogen atmosphere from room temperature to 700 °C and annealed for 2 h. The obtained products were named LPCs, where s is the annealing temperature. The carbon samples were obtained finally through HCl acid etching, washed with deionized water, and dried in an air-flow oven.

### Materials Characterization

Thermogravimetric analysis coupled with mass spectrometry and IR spectra (TGA–DSC-MS-FTIR) was performed using a NETZSCH simultaneous thermal analyzer (STA 449 F3, Germany) together with a NETZSCH mass spectrometer (QMS 403, Germany) and a NETZSCH FTIR spectrometer (Nicolet iS20, Germany) at a heating ramp of 10 °C min^–1^ in Ar atmosphere. The microstructure of NSLPCs was examined by a Talos F200S FE-TEM (FEI Thermo Scientific, USA). Field emission scanning electron microscopy (FE-SEM) images were acquired on a SU8220 scanning electron microscope (HITACHI, Japan). X-ray photoelectron spectroscopy (XPS) analysis was conducted on an ESCA lab 250Xi photoelectron spectrometer (Thermo Fisher Scientific, USA). The vacancy defect structure of NSLPCs was evaluated by an EMXplus-10/12 electron paramagnetic resonance (Bruker, Germany). XRD patterns were collected on a D8 Advance X-ray diffractometer (Bruker, Germany) with Cu Kα radiation (λ = 1.5406 Å). The specific surface area and pore textures of carbon samples were analyzed by an ASAP 2460 N_2_ adsorption/desorption isotherm analyzer (Micromeritics, USA) at 77 K. Raman spectra were tested by a Lab RAM HR micro-Raman spectrometer (HORIBA Jobin Yvon, France) using a 532 nm laser with a filter of 3%. The Fourier transform infrared spectroscopy (FTIR) of samples was collected on an IS50R Fourier spectrometer (Thermo Fisher Scientific, USA).

### Electrode Preparation and Electrochemical Measurements

#### Fabrication of NSLPCs Electrodes

The working electrodes of the half-cells were prepared by mixing active materials (NSLPCs, LPC-700), conductive carbon black, and sodium carboxymethylcellulose (2 wt%) (CMC) on copper foil with a mass ratio of 8:1:1. The mass load of active materials on each working electrode was about 0.8 mg cm^–2^. The electrochemical performance was tested in a 2032-coin cell with K metal as the counter electrode and glass fiber (GF/B, Whatman) as the separator. 3 M potassium bis(fluorosulfonyl)imide (KFSI) in ethylene glycol dimethyl ether (DME) solvent was used as the electrolyte.

#### Electrochemical Measurements

The galvanostatic charge/discharge and galvanostatic intermittent titration technique (GITT) tests were conducted in the voltage range of 0.01–3.00 V using a Neware battery test system (Neware, Shenzhen, China). Cyclic voltammetry (CV) curves were measured by a VMP3 electrochemical workstation (Bio-logic, France). The electrochemical performance of NSLPC-700//AC (YP-50F) potassium-ion hybrid capacitors (PIHCs) in the voltage range of 0.01–3.6 V was explored. The mass load of the NSLPC-700 anode was approximately 0.8–1 mg cm^–2^ and that of the active material of AC was approximately 1.4–2 mg cm^–2^. The mass ratio of negative to positive electrodes was approximately 1:1.5–3. The NSLPC-700 and YP-50F electrodes were pre-cycled for five cycles (50 mA g^–1^) prior to the assembly of PIHCs.

## Results and Discussion

### Synthesis Mechanism of NSLPC

**Fig. 1:**
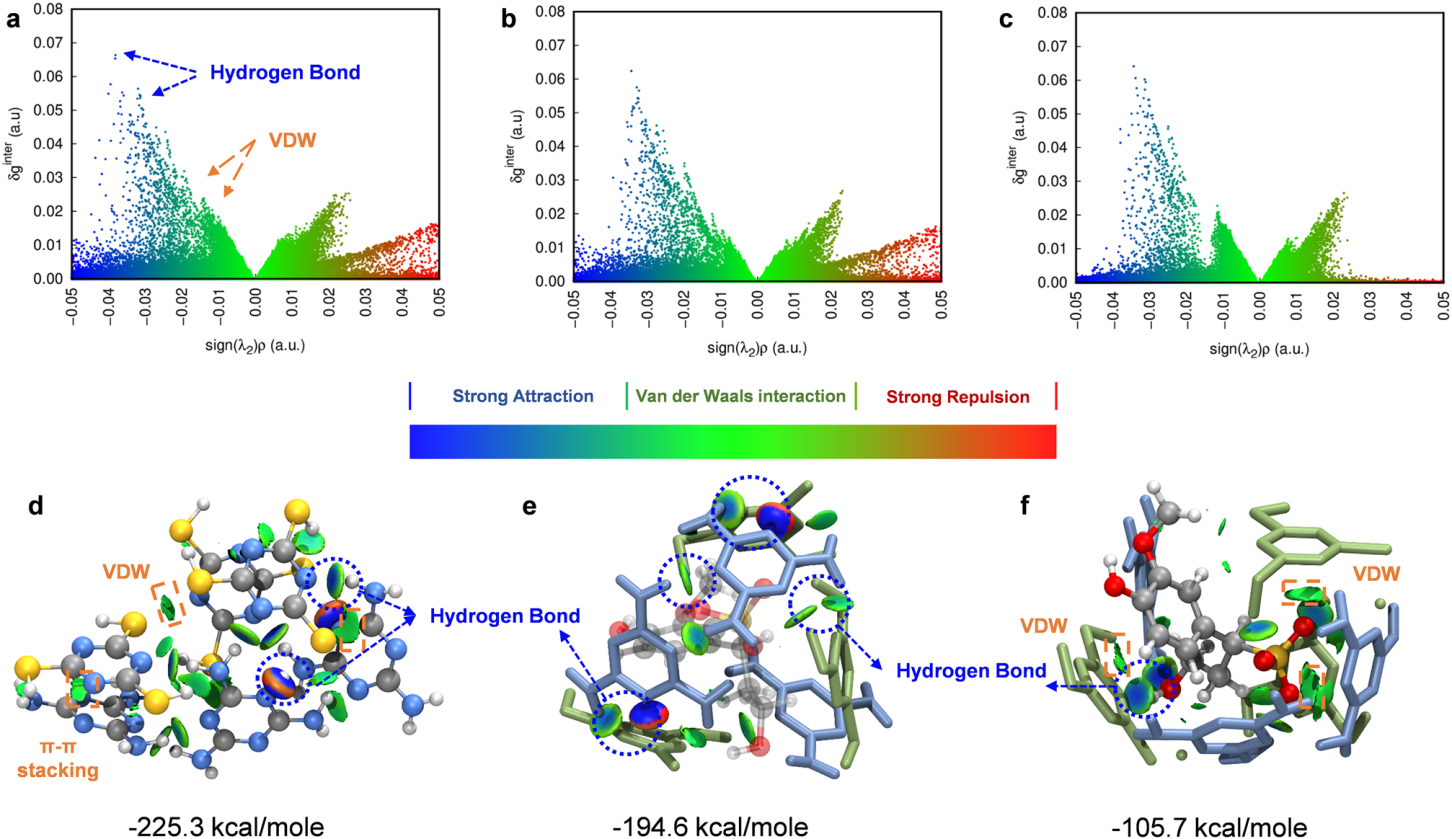
**a-c** Scatter graph and **d-f** isosurface (value = 0.01 electrons/Bohr.^3^) of independent gradient model based on Hirshfeld partition (IGMH) for unveiling the interaction between MA and TCA with/without the interference of LS model molecule, respectively (MA + TCA assembly in (**d**) CPK model, C-gray, N-blue, H-white, S-yellow; MA + TCA + LS assembly in (**e, f**) licorice model, MA-blue, TCA-green)

A molecular simulation method was used to study the interactions between MA + TCA (MA and TCA, respectively, represent melamine and trithiocyanuric acid) and sodium lignosulfonate (LS). The independent gradient model based on Hirshfeld partition (IGMH) was used to describe electron density gradient across the supermolecules and plotted to unveil noncovalent interactions. The blue peaks and isosurfaces (Fig. 1a) among molecules exhibited strong hydrogen bonding (N‧‧‧H-N and N‧‧‧H–S) [39, 40] interactions between MA and TCA as well as between themselves, accompanied by an energy of − 225.3 kcal mole^−1^ (Fig. 1d). The green region indicates that van der Waals (VDW) interactions including π-π stacking also contributed to the assembly of MA + TCA. The interaction between MA and TCA was weakened by LS indicated by fewer peaks in Fig. 1b and a smaller isosurface area in Fig. 1e. Interestingly, their interaction was weakened by LS to − 194.6 kcal mol^−1^ (Fig. 1c, f) due to the strong interaction between LS and MA + TCA (− 105.7 kcal mol^−1^), resulting in the formation of supermolecule/lignin precursors.

**Fig. 2:**
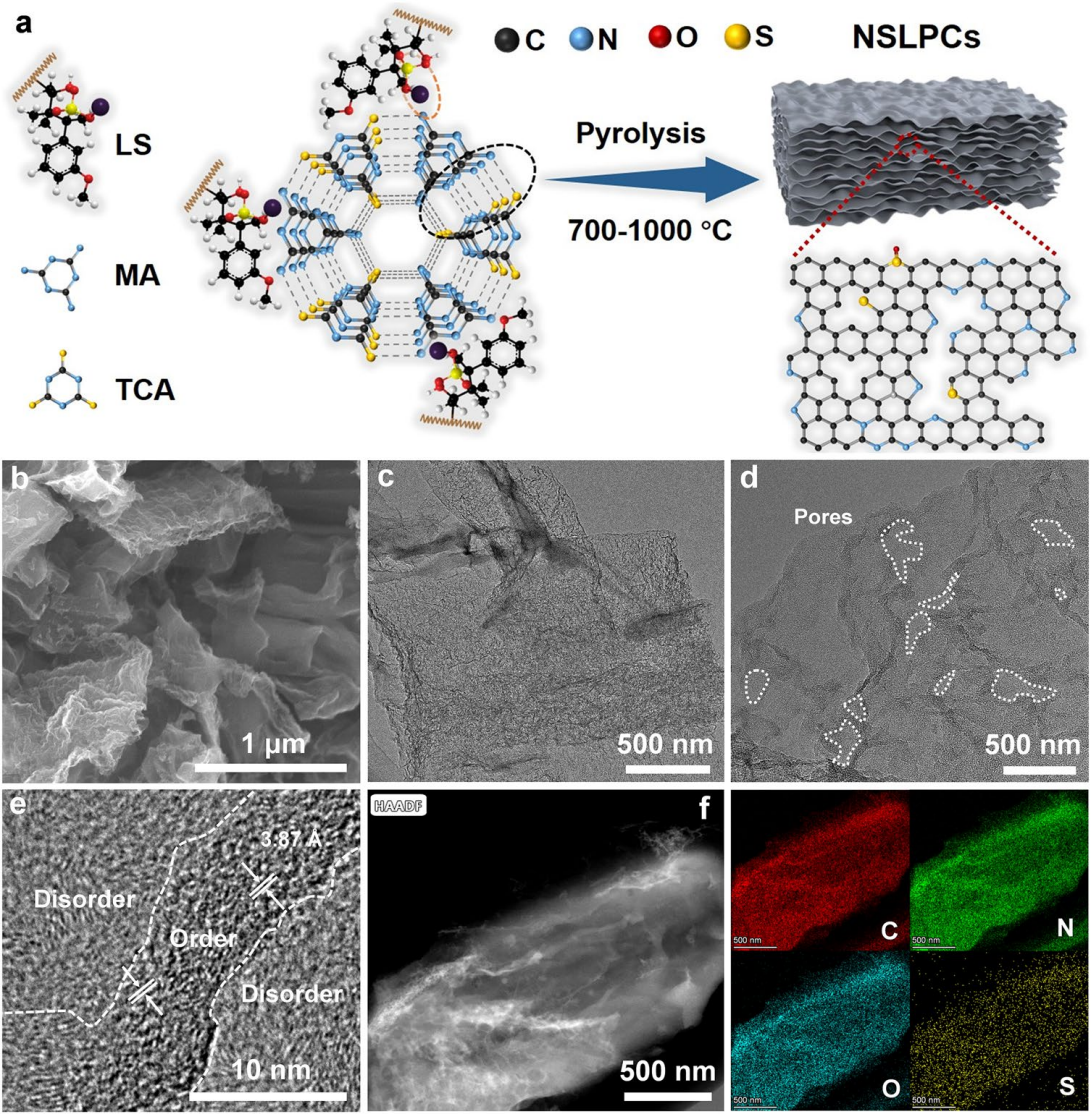
**a** Schematic illustrating the preparation of NSLPCs. **b** SEM image of NSLPC-700. (**c-e**) TEM images of NSLPC-700. **f** EDS elemental mapping of NSLPC-700

The melamine thiocyanurate supermolecule/lignin precursor (supermolecule was formed by the interactions of hydrogen bonds [39, 40]) was obtained by a pyrolysis carbonization strategy with sodium lignosulfonate (LS), melamine (MA) and trithiocyanuric acid (TCA) supermolecule [22]. Afterward, the precursor (supermolecule/LS) was pre-carbonized at 550 °C for 1 h and then heated up to 700–1000 °C for 2 h to obtain carbon products (Fig. 2a). The final N/S-doped lignin-derived porous carbons (NSLPCs) were then obtained by dilute HCl acid and water washing (where carbon materials were denoted as NSLPC-X at the annealing temperatures of X °C). The SEM images of NSLPC-700 are displayed in Fig. 2b, c, which exhibits a nanosheet structure formed due to the good dispersion effect of supermolecule. Similarly, NSLPC-800, NSLPC-900, and NSLPC-1000 also showed nanosheet structures (Fig. S1a-f). Notably, NSLPC-700 showed a porous and short-range ordered structure with an average interlayer spacing of ~ 3.87 Å (Fig. 2d, e). The C/N/O/S elements were uniformly distributed on the skeletons of NSLPC-700 and NSLPC-800 (Figs. 2f and S1g).

**Fig. 3:**
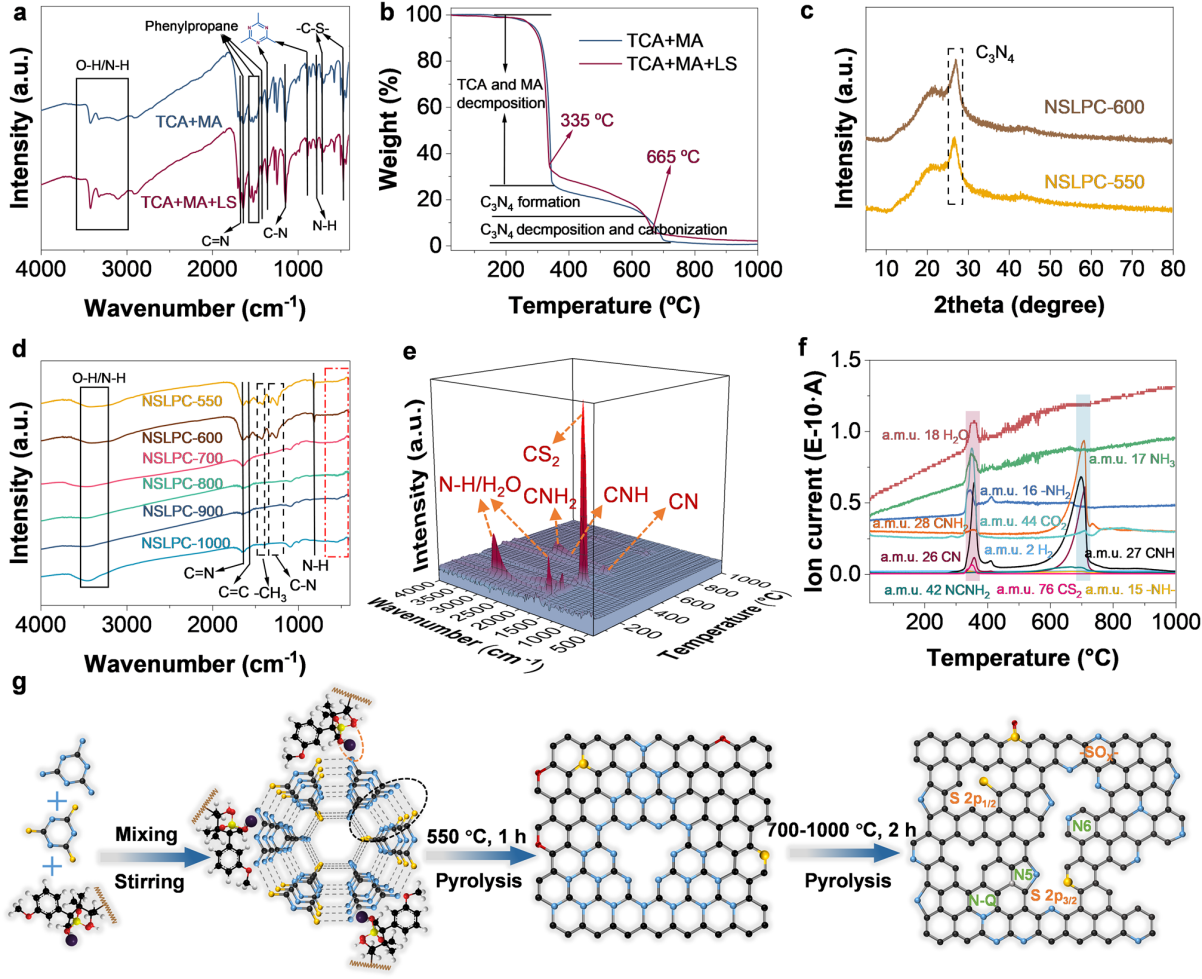
**a** FTIR spectra of TCA + MA and TCA + MA + LS. **b** TGA curves of TCA + MA and TCA + MA + LS. **c** XRD patterns of NSLPC-550 and NSLPC-600. **d** FTIR spectra of NSLPC-550, NSLPC-600, NSLPC-700, NSLPC-800, NSLPC-900, NSLPC-1000. **e** TGA-FTIR spectra. **f** TGA-MS spectra of the TCA + MA + LS along with the carbonization temperature. **g** Pyrolysis reaction mechanism

To further explore the pyrolysis mechanism of the supermolecule/LS precursor, we studied the structural evolution during the transformation from precursor molecules to NSLPCs by thermogravimetric analysis coupled with mass spectrometry and IR spectra (TGA-MS and TGA-FTIR). The FTIR curves of both TCA + MA and TCA + MA + LS precursors are shown in Fig. 3a. The infrared absorption peaks near 900 and 1370 cm^‒1^ corresponded to the triazine ring. Meanwhile, the infrared absorption peaks of C-S (~ 490 and 704 cm^‒1^) [41], N–H (~ 760 cm^‒1^), C-N (~ 1200 cm^‒1^), and C = N (~ 1650 cm^‒1^) bonds were clearly observed. The enhanced absorption peaks near 1615, 1495, and 1410 cm^–1^ (phenylpropane monomer) [42] proved that LS had been successfully assembled in the as-synthesized supermolecule. Notably, the color gradually changed from bright yellow to dark yellow in the preparation process of the precursors, further confirming the introduction of LS (Figs. S2 and S3). Meanwhile, the FTIR spectra (Fig. S4) and X-ray diffraction (XRD) spectra (Fig. S5a) of TCA and MA were different, which reveals that the introduction of LS resulted in a significant change in the XRD peaks from 25° to 30°, confirming the existence of their interaction and the formation of a novel lignin/supramolecule precursor (Fig. S5b).

The pyrolysis process of TCA + MA + LS (Fig. 3b) was divided into three stages (25–335 °C, 335–665 °C, and 665–1000 °C). In the first stage, weight loss (67%) was mainly attributed to the decomposition of TCA and MA, resulting in the formation of fragments (N–H, C = N, -CH_2_-, etc.) and gaseous molecules (CO_2_, H_2_O, etc.), accompanied by the gradual loss of -C-S- bonds (Fig. S6a, b). In the second stage, the weight loss (25%) was mainly caused by the formation of C_3_N_4_ intermediate products (Fig. 3c) covalently bonded with carbon skeleton derived from lignin, accompanied by volatilized fragments (C = N, -NH_2_, CNH, N–H, and CNH_2_) (Fig. 3d) and molecules (NH_3_, CO_2_, CS_2_) (The IR absorption peaks of CO_2_ and CS_2_ were observed near 2250 and 1510 cm^–1^, respectively [43].) (Figs. 3e-f, S6c-d, S7a-b and S8). In the third stage, weight loss was attributed to the volatilized fragments such as C-N, C-NC, C = N (Fig. S9), CNH, CNH_2_, NCNH_2_ (Fig. 3e, f), and CO_2_ (Fig. S6e-h). Meanwhile, C-N (~ 1200 cm^–1^), N–H (~ 760 cm^–1^), and C-S (~ 490 and 704 cm^–1^) bonds in NSLPC-700, NSLPC-800, NSLPC-900, and NSLPC-1000 slightly cracked (Fig. 3d). For this reason, the carbonization of lignin and decomposition of small amounts of nitrogen-containing molecules and gases led to a weight loss of 3% in this stage.

Notably, supermolecular precursors experienced morphological change from irregular blocks (Fig. S10) to nanosheets (Fig. S11) from 550 to 700 °C (Fig. 3g). This was caused by the decomposition reaction of C_3_N_4_ and molecular fragments and gases (Fig. S12) that endowed superb edge nitrogen doping (pyridinic-N and pyrrolic-N) configurations and abundant edge defect adsorption sites for K^+^. The pyrolysis mechanism of TCA + MA (Fig. 3b) was similar to that of TCA + MA + LS, but no carbonaceous material was formed at 1000 °C. Therefore, the introduction of TCA + MA in the carbonization process would induce the formation of nanosheet morphology and abundant defects.

### Physicochemical Characterizations of NSLPC

**Fig. 4:**
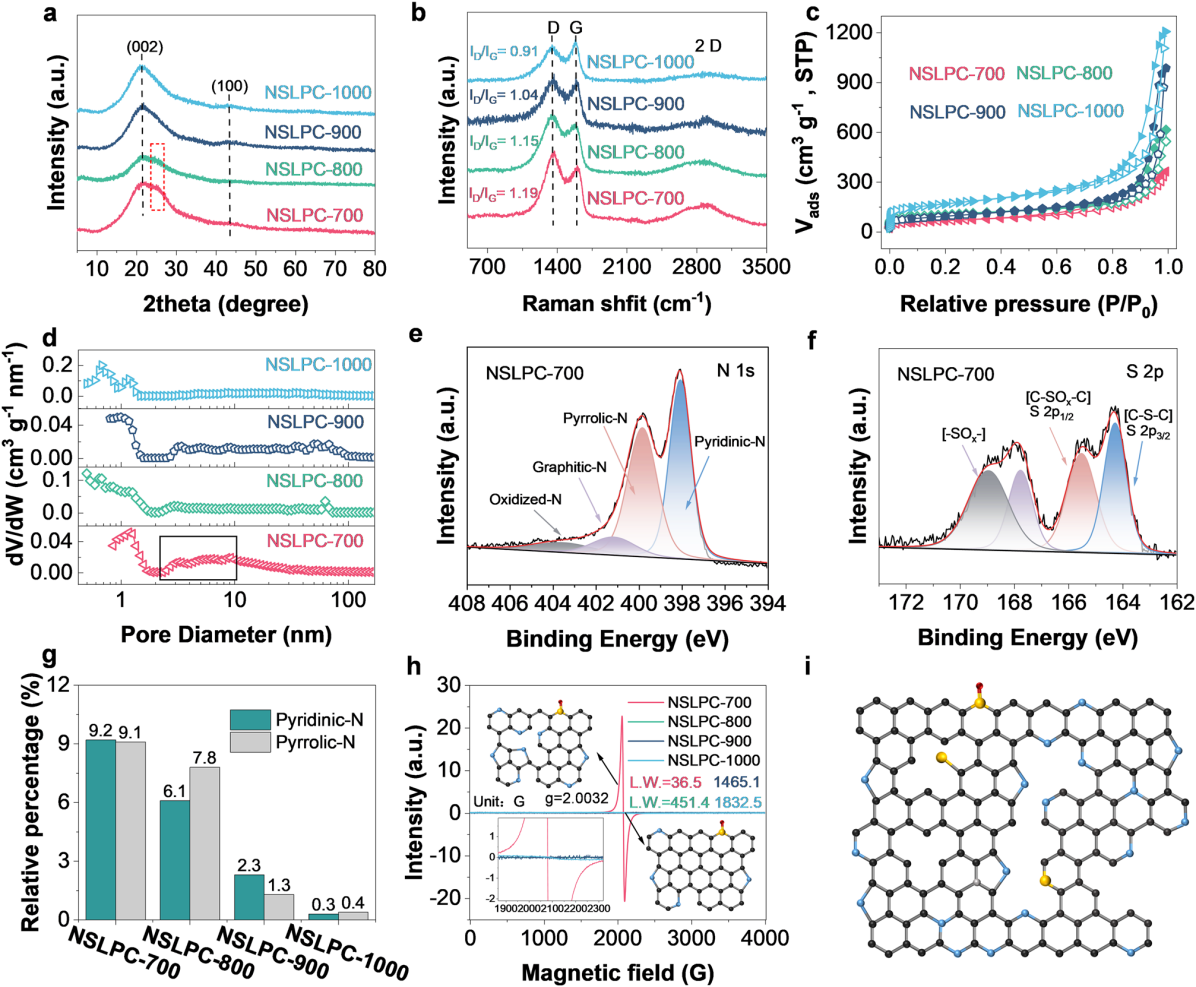
**a** XRD patterns. **b** Raman spectra. **c** N_2_ adsorption/desorption isotherms, and **d** pore size distribution of the NSLPCs. High-resolution **e** N 1* s* and **f** S 2*p* XPS spectra of the NSLPC-700. **g** XPS survey calculated the elemental contents of the pyridinic-N and pyrrolic-N. **h** EPR spectra of NSLPCs. **i** Schematic illustrating a heteroatom-doping configuration in NSLPC

The X-ray diffraction patterns (XRD) of NSLPC-700, NSLPC-800, NSLPC-900, and NSLPC-1000 are shown in Fig. 4a. The broad diffraction peaks near 22.4° and 43° (002 and 100 plane) indicate that NSLPCs were all highly disordered carbon materials [15, 44]. Particularly, NSLPC-700 still exhibited a weak diffraction peak near 26° belonging to C_3_N_4_ [45]. Some fragments of NSLPC-700 were observed to be derived from C_3_N_4_, resulting in high nitrogen doping. Thus, our preliminary speculation is that NSLPC-700 contained some fragments from C_3_N_4_ after being doped with nitrogen. The Raman spectra are shown in Fig. 4b. Two characteristic diffraction peaks were observed near 1380 cm^–1^ (D band) and 1589 cm^–1^ (G band), the former of which was ascribed to *sp*^3^ hybridized carbon (A_1g_ vibrational model) and the latter to the ordered *sp*^2^ hybridized carbon (*E*_2g_ vibrational model) [46, 47]. The intensity ratio of *I*_D_/*I*_G_ gradually decreased with the increase in the pyrolysis temperature (*I*_D_/*I*_G_ values for NSLPCs were 1.19, 1.15, 1.04, and 0.91, respectively). NSLPC-700 had the highest *I*_D_/*I*_G_ value, indicating its highest degree of defects. The Brunauer–Emmett–Teller (BET) surface areas and pore size distribution of NSLPCs were evaluated by nitrogen adsorption/desorption isotherms [48–50]. As shown in Fig. 4c, the N_2_ adsorption/desorption isotherms of NSLPCs showed a sharp increase under low adsorption pressure (P/P_0_ < 0.01), indicating that NSLPCs contained plenty of micropores. The H3-type hysteresis loop under medium adsorption pressure (0.45 < P/P_0_ < 0.9) and the dramatic rise under high adsorption pressure (0.9 < P/P_0_ < 1) indicate the presence of mesopores and macropores [48]. The pore size of NSLPCs was mainly distributed in the range of micropores (0–2 nm) and mesopores (2–50 nm) (Fig. 4d) [31, 51]. Meanwhile, the specific surface areas (SSAs) of NSLPC-700, NSLPC-800, NSLPC-900, and NSLPC-1000 were 229, 362, 322, and 589 cm^2^ g^–1^, respectively (Table S1). NSLPC-700 was also enriched with micropores (0.7–2 nm) and mesopores (2–10 nm), which provides efficient diffusion channels for K^+^. The surface composition and chemical environments of NSLPCs were further analyzed by X-ray photoelectron spectrum (XPS). C, N, O, and S elements were identified in NSLPCs (Fig. S13). The atomic ratios of N elements for NSLPC-700, NSLPC-800, NSLPC-900, and NSLPC-1000 were 21.6, 17.0, 7.2, and 2.4 at%, respectively. Meanwhile, the atomic percentages of S elements were 0.8, 0.6, 0.6, and 1.2 at%, respectively (Table S2). The NSLPC doped with high contents of N and S elements could improve the storage performance of K^+^ since these heteroatoms were able to induce abundant edge defect sites. The high-resolution XPS spectra of C, N, O, and S elements in NSLPCs were further analyzed. The C 1* s* spectra were fitted into five peaks corresponding to C = C/C–C (~ 284.8 eV), C-N (~ 286.1 eV), C-S (~ 287.4 eV), C = O (~ 289.1 eV), and O-C = O (~ 291.3 eV). The O 1* s* were divided into three bonding configurations: C = O (~ 531.4 eV), C–OH/C–O–C (~ 532.3 eV), and − COOH/H_2_O (~ 534.0 eV). The N 1* s* XPS spectra were deconvoluted into three distinct parts, namely pyridinic-N (~ 398.2 eV), pyrrolic-N (~ 399.5 eV), graphitic-N (~ 400.4 eV), and oxidized-N (~ 402.2 eV). The S 2*p* was divided into three types, namely C–S–C (~ 163.8 eV), C–SO_X_–C (X = 2–4, ~ 164.9 eV), -SO_X_-, (~ 167.4/168.7 eV) (Tables S3 and S4). The high-resolution XPS spectra of N and S elements of NSLPC-700 are shown in Fig. 4e, f, respectively. NSLPC-700 contained a significant amount of pyridinic and pyrrolic nitrogen dopants, along with sulfur dopants in the form of C–S–C and C–SO_X_–C. The existence of pyridinic and pyrrolic nitrogen dopants created a large number of edge-type defects [22], increasing the adsorption ability of K^+^ and improving the storage performance of potassium ions. The C-S-C and C-SO_X_-C bonds also induced edge defects and improved wettability as well as electrochemical performance [43, 52, 53]. The detailed analysis of high-resolution XPS spectra of other C/N/O/S elements in NSLPC-700, NSLPC-800, NSLPC-900, and NSLPC-1000 are shown in Figs. S14-S16. The doping on the edge of NSLPC-700 reached 18.3 at%, which indicates its high ability to adsorb K^+^ (Fig. 4g) [4]. Therefore, combining high-edge defects and highly disordered structures, we propose a “defect in disorder” terminology to describe the importance of creating defect structures in amorphous carbons for storing K^+^. The edge nitrogen doping effect of NSLPCs was further investigated by electron paramagnetic resonance spectroscopy (EPR) (Fig. 4h). The EPR spectra of NSLPC-700, NSLPC-800, NSLPC-900, and NSLPC-1000 exhibited classical Lorentzian signal lines centered at g = 2.0032. NSLPC-700 exhibited a narrower line width (36.5 G) than NSLPC-800, NSLPC-900, and NSLPC-1000 (451.4, 1465.1, and 1832.5 G, respectively), indicating that the unpaired electrons of NSLPC-700 were more localized. The localized unpaired electrons of NSLPC-700 indicate that NSLPC-700 experienced abundant edge nitrogen doping (Fig. 4i) [54]. The unique ultra-high edge-nitrogen-doped structure of NSLPC-700 agrees well with the XPS and Raman results. Therefore, the proposed “defect in disorder” terminology facilitates the understanding of edge effects in amorphous carbons for efficient storage of K^+^.

### Potassium-ion Storage Performance

**Fig. 5:**
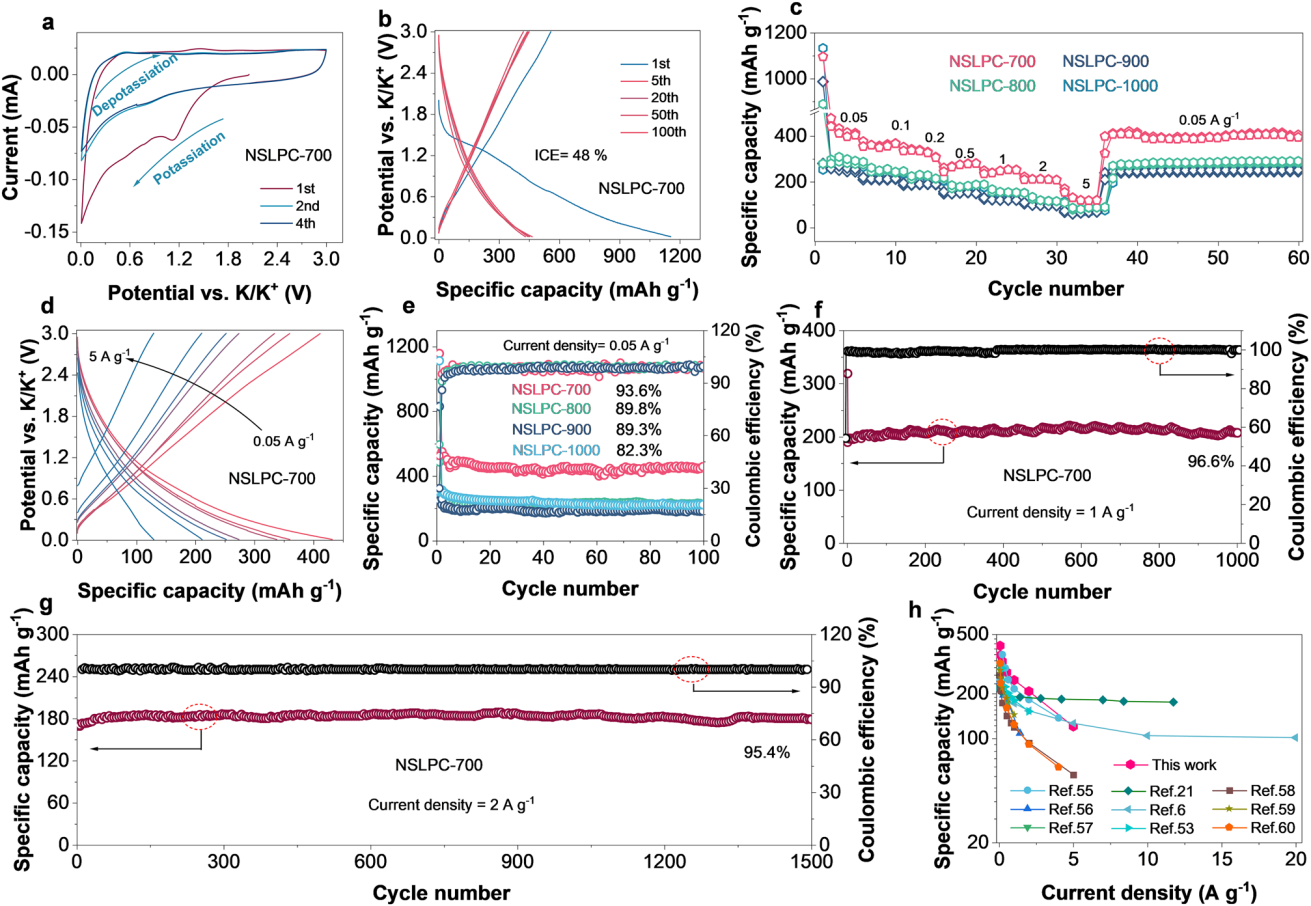
**a** CV curves of the NSLPC-700 anode at a scan rate of 0.1 mV s^−1^. **b** GCD curves of NSLPC-700 at 0.05 A g^−1^. **c** Rate performances of NSLPCs. **d** GCD curves at different current densities. **e** GCD cycling performances of NSLPCs at 0.05 A g^−1^ over 100 cycles. **f** Long-term GCD cycling performance of NSLPC-700 at 1 A g^−1^ over 1000 cycles. **g** Long-term cycling performance of NSLPC-700 at 2 A g^−1^ over 1500 cycles. **h** Specific capacities of the NSLPC-700 compared with the recently reported amorphous carbon anodes

The NSLPCs with different degrees of defects were further used as anodes to store K^+^. The formation of cathodic peak in the cyclic voltammetry (CV) curve near 1.2 V was mainly attributed to the emergence of solid electrolyte interface (SEI) (Fig. 5a) [61]. After the first cycle, the CV curves well overlapped with each other, which demonstrates its superior reversibility. The galvanostatic charge–discharge (GCD) curves of the NSLPC-700 anode at 1, 5, 20, 50, and 100 cycles were highly overlapping (Fig. 5b), indicating its excellent reversibility. The reversible capacities of the NSLPC-700, NSLPC-800, NSLPC-900, and NSLPC-1000 anodes at 0.05 A g^–1^ were initially evaluated (Fig. S17) to be 419, 302, 254, and 276 mAh g^–1^, respectively. Notably, the NSLPC-700 anode demonstrated excellent rate performance, with capacities being 419, 362, 329, 278, 246, 207, and 117 mAh g^–1^ at the current densities of 0.05, 0.1, 0.2, 0.5, 1, 2, and 5 A g^–1^, respectively (Fig. 5c). Its reversible capacity reached 418 mAh g^–1^ when current density was reduced to 0.05 A g^–1^. This was mainly attributed to the unique “defect in disorder” structure that substantially enhanced the ability to adsorb K^+^. It was also observed that the capacity tended to decay as current density was increased from 0.05 to 5 A g^–1^ (Fig. 5d). The long-term cycling tests of NSLPC-700, NSLPC-800, NSLPC-900, and NSLPC-1000 anodes at 0.05 A g^–1^ are shown in Fig. 5e. The NSLPC-700 anode had the best capacity retention of 93.6% and a reversible capacity of 402 mAh g^–1^ after 100 cycles, while the NSLPC-800, NSLPC-900, and NSLPC-1000 anodes showed capacity retentions of 89.8%, 89.3%, and 82.3% at 0.05 A g^–1^, respectively. Meanwhile, the unique “defect in disorder” structure enabled the NSLPC-700 anode to achieve a high initial coulomb efficiency (ICE) of 48% (compared with 21% for NSLPC-800, 30% for NSLPC-900, and 25% for NSLPC-1000), which was superior to previously reported heteroatom-doped carbon anodes (Table S5). The capacity retention of the NSLPC-700 anode was 96.6% and 95.4% after 1000 and 1500 cycles at 1 and 2 A g^–1^, respectively (Fig. 5f, g). Meanwhile, the NSLPC-800, NSLPC-900, and NSLPC-1000 anodes also showed well-overlapped CV curves in the potassium-ion half-cells (Figs. S18a, c, S21a, b and S23a, b) and excellent cycling stability at 1 and 2 A g^‒1^ (Figs. S18b, S19, S20, S21c-d, S22, and S23c).

The K^+^ storage electrochemical performance of NSLPC-700 was superior to that of other NSLPC samples due to its most abundant edge defects. Interestingly, the blank control sample of lignin-derived porous carbon (LPC-700) showed a lumpy morphology (Fig. S24). In contrast, NSLPC-700, NSLPC-800, NSLPC-900, and NSLPC-1000 all showed nanosheets arising from the decomposition of covalently bonded C_3_N_4_ in the carbon skeleton. This preferential decomposition not only induced abundant nitrogen doping but also promoted the formation of the nanosheet morphology with abundant mesopores, which was conducive to the diffusion of K^+^.

LPC-700 is also an amorphous carbon with an I_D_/I_G_ value of 1.06 (Fig. S25). The low degree of edge defects and lumpy structure resulted in the inferior potassium-ion storage in the LPC-700 anode (131 mAh g^–1^) (Fig. S26). Hence, NSLPC-700 exhibited high capacity and high-rate capability. The potassium-ion storage performance of NSLPC-600 (28 mAh g^–1^) was also much poorer than that of NSLPC-700 as it was incompletely carbonized at 600 °C (Fig. S27a, b). The incomplete carbonization of NSLPC-600 also suggested a significant fraction of C_3_N_4_ in the carbon skeleton (the appearance of C_3_N_4_ sharp diffraction peak near 26° (Fig. 3c)), leading to the poor electronic conductivity of NSLPC-600. Notably, potassium-ion half-cells assembled by the NSLPC-700 anode showed superior performance to that in other studies (Fig. 5h and Table S6). Therefore, “defect in disorder” strategy proposed in this study for designing NSLPC-700 could give rise to both high capacities and high-rate capabilities.

### Potassium Storage Mechanisms

**Fig. 6:**
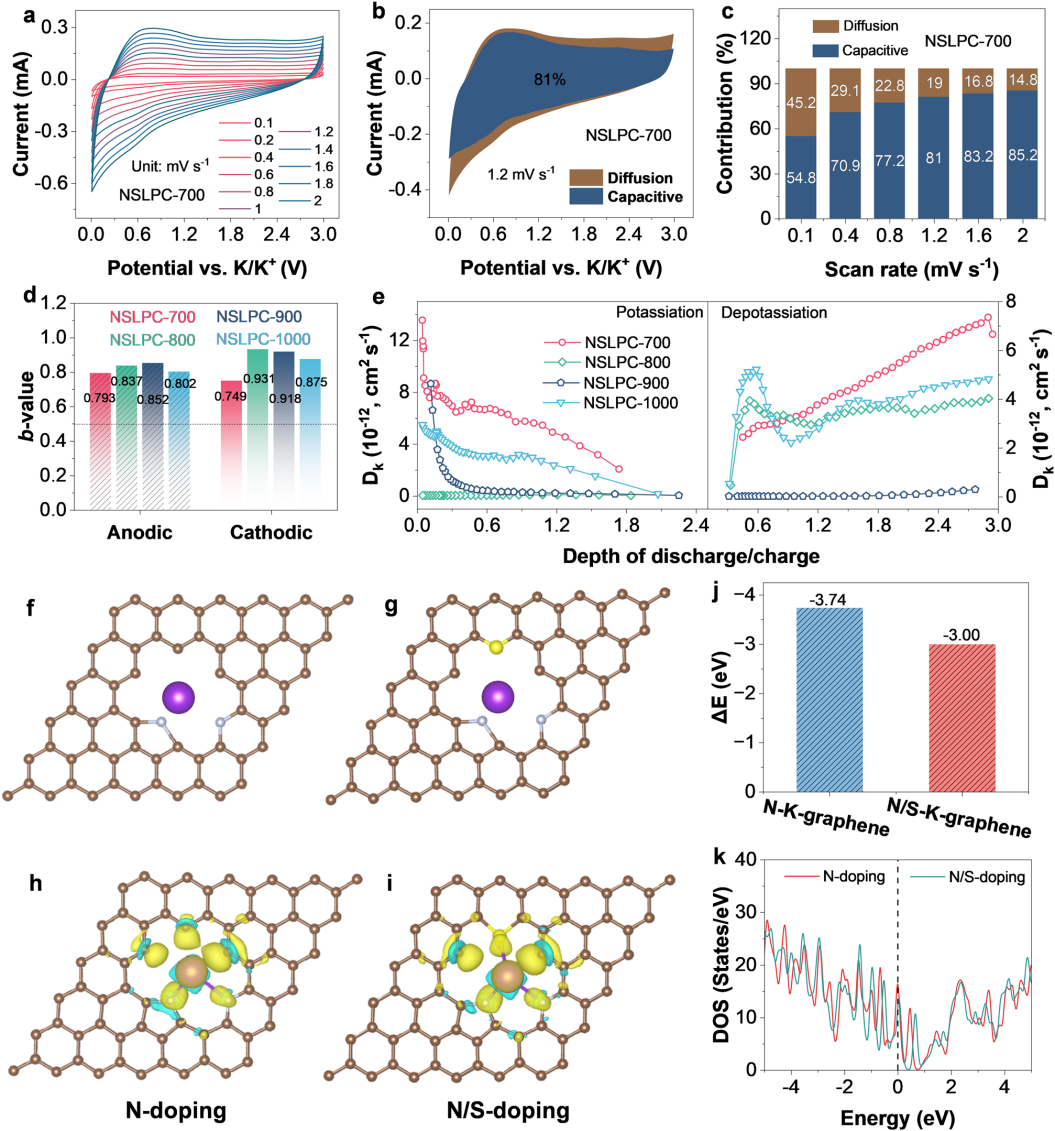
**a** CV curves of the NSLPC-700 anode at different scan rates. **b** Capacitive contributions of NSLPC-700 anode at a scan rate of 1.2 mV s^−1^. **c** Capacitive contributions of NSLPCs anode as a function of scan rate. **d** Comparison of *b* values of NSLPC samples. **e** K^+^ diffusion coefficient of NSLPCs obtained via the GITT technique during depotassiation and potassiation processes. Front views of optimized models with a single K adsorbed on the **f** N-doped, **g** N/S-doped defective graphene sheet and their corresponding **j** binding energy (ΔE). **h, i** Calculated difference charge density of K adsorbed onto different structures. **k** Density of states (DOS) of the N-doped, N/S-doped graphene

To further understand the charge storage kinetics of host, the potassium-ion storage ability of the NSLPC-700 anode was further characterized by CV, galvanostatic intermittent titration technique (GITT), and theoretical calculations. As displayed in Fig. 6a, the shape of the CV curve was basically constant with the continuous increase in scan rate, indicating the good stability of NSLPC-700. The source of capacity was quantitatively separated into two sections, *i.e.,* diffusion-controlled intercalation process and surface capacitance-controlled process [7]. The capacitance-controlled contributions of NSLPC-700, NSLPC-800, NSLPC-900, and NSLPC-1000 were achieved at 81.0%, 80.8%, 84.2%, and 80.0% at a scan rate of 1.2 mV s^–1^, respectively (Figs. 6b and S28a-c). The surficial capacitance control capacity contribution of NSLPC-700, NSLPC-800, NSLPC-900, and NSLPC-1000 anodes gradually increased with the increase in scan rate. Their capacitance contributions to capacitance-controlled process at a scan rate of 2 mV s^–1^ reached 85.2%, 85.4%, 88.0%, and 88.8%, respectively (Figs. 6c and S28d, f). Interestingly, the NSLPC-700 anode with the best potassium storage performance did not demonstrate the most capacitance-controlled capacity contribution because a diffusion-controlled process was caused by the adsorption of K^+^ on their cross-linked carbon skeleton structure. The *b* value was calculated to indicate the potassium-ion storage mechanism of NSLPCs. While *b* = 1 means the diffusion-controlled process, the surface capacitance-controlled process should possess a *b* value of 0.5 [26]. The *b* values fitted to the anode and cathode peaks of the NSLPC-700 anode were 0.783 and 0.749, respectively (Fig. 6d). We further evaluated the K^+^ diffusion in NSLPCs using the Galvanostatic Intermittent Titration Technique (GITT) (Fig. S29) [62]. The K^+^ diffusion coefficient of the NSLPC-700 anode was the highest in both potassiation and depotassiation processes (Fig. 6e).

To further analyze the synergistic effect of the co-doping of N and S on the adsorption of K^+^, we systematically analyzed the differential charge adsorption models and the density of states (DOS) for N-doped and N/S co-doped defective graphene using density functional theory (DFT) [63]. The density of states for blank C/N/S elements is shown in Fig. S30a, b. The models of N-doped graphene and N/S-doped graphene with potassium adsorbed are shown in Fig. 6f, g, while their adsorption energies were calculated to be 3.74 and 3.0 eV, respectively (Fig. 6j). Paradoxically, the adsorption energy was even lower than that of N doping after N/S doping, which was different from the results obtained in most recent reports [41, 52], indicating that S doping did not contribute to the adsorption of K^+^. S dopants (C-S-C) created specific defect sites, which was also a prerequisite for the formation of edge defects in amorphous carbons. It has recently been reported that S doping can enhance the performance of potassium-ion storage by improving the wettability of the electrode surface [43, 52, 53].

Whichever dopant is applied, the carbon-vacancy defects are crucial for the storage of K^+^, which is in accordance with our previous study [31]. The above results prove that abundant edge defects could significantly provide adsorption sites. Therefore, S dopants and edge nitrogen dopants play similarly important roles in creating defect sites.

Meanwhile, the same conclusion was obtained for the differential charges of N-doping and N/S doping, that is, N/S doping did not effectively promote the adsorption of K^+^ (the electron did not lead to a significant change for N/S co-doping) (Fig. 6h, i). The co-doping of N/S elements did not increase significantly at the Fermi energy level, indicating that S doping did not improve the conductivity of the carbon material (Fig. 6k). Nevertheless, different doping configurations contributed to the storage of K^+^ through different chemical bonding or specific adsorptions. Therefore, we further explored the K^+^ adsorption mechanism of NSLPCs with a “defect in disorder” structure during potassium storage using ex situ XPS and Raman techniques.

**Fig. 7:**
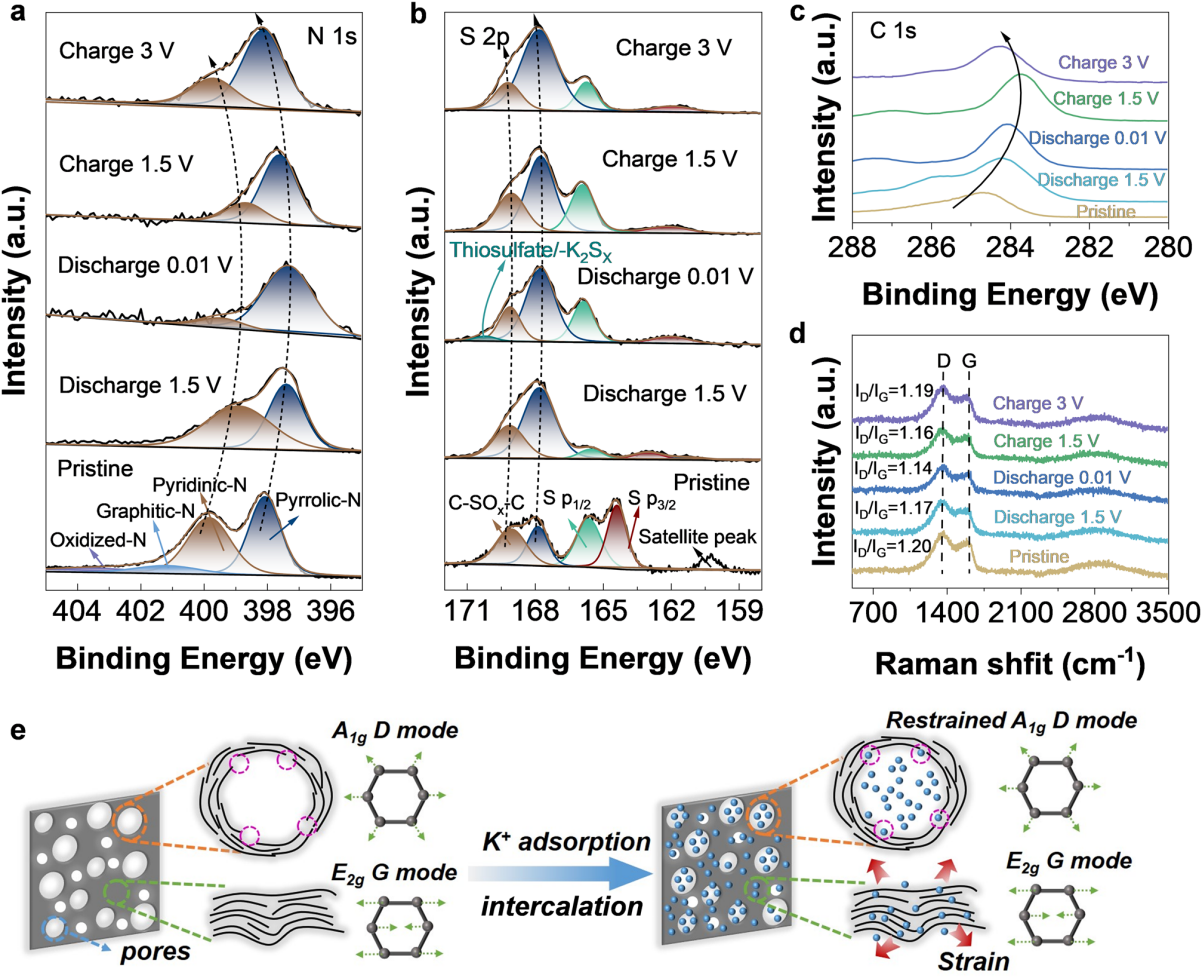
Potassium-ion storage mechanism study of the NSLPC-700. Ex situ XPS **a** N 1*s*, **b** S 2*p* and **c** C 1*s* spectra of NSLPC-700 anode **d** Ex situ Raman spectra of NSLPC-700 anode. **e** Schematic illustrating the potassium-ion storage in amorphous defect-rich carbons

The GCD curves of NSLPC-700 anode at different charge and discharge states (pristine, 1.5, 0.01, 1.5, 3 V) are shown in Fig. S31a. The NSLPC-700 anode contained C/N/O/S/K elements at different states (Fig. S31b). The high-resolution N 1*s* spectra of NSLPC-700 are shown in Fig. 7a, where pyridinic-N (398.2 eV) and pyrrolic-N (399.7 eV) witnessed blueshift phenomenon as the potential decreased, resulting from the fact that “defect in disorder” structure adsorbs a large amount of reduced K^+^ on these sites at low potentials [43]. As the potential gradually increased to 3 V, the redshift phenomenon occurred again, and the binding energy was reduced to its initial values at 398.2 and 399.7 eV, respectively [64]. Notably, the high-resolution S 2*p* spectra showed the same phenomenon as the N 1*s* one (Fig. 7b). There was a slight blueshift and redshift with decreased and increased potentials. The high-resolution S 2*p* and N 1*s* XPS spectra differed in that S 2*p* adsorbed K^+^ forming Thiosulfate/-K_2_S_X_ at 0.01 V [65]. The above phenomena demonstrate that “defects in disorder” structure could reversibly adsorb large amounts of K^+^ through chemical bonding with sulfur and nitrogen dopants, thus improving potassium storage kinetics. Meanwhile, the XPS high-resolution spectra of K 2*p* and C 1*s* shown in Figs. S32 and 7c demonstrate that the NSLPC-700 anode was highly reversible. The *ex situ* Raman spectra of the NSLPC-700 anode are shown in Fig. 7d, where the values of *I*_D_/*I*_G_ decreased and then increased with the change of potential (from 1.20, 1.17, 1.14, 1.16 to 1.19) and it was attributed to the “defects in disorder” structure in the NSLPC-700 anode which could adsorb a large amount of K^+^ at low potentials, thus occupying a large number of edge defect sites and resulting in the decrease in the defect degree and the *I*_D_/*I*_G_ value. Simultaneously, as the potential increased, K^+^ ions were desorbed, and *I*_D_/*I*_G_ increased to the value almost the same as that at the initial potential, which provided more evidence that the potassium storage process was highly reversible. Notably, the intensity of the G-peak shifted from strong to weak with the change in potential and thiophene/-K_2_S_X_ emerged in the XPS spectrum (S 2*p*), proving that a small amount of K^+^ were desorbed in the intercalation/deintercalation reaction [66]. The NSLPC-700 anode occupied defect sites after the adsorption of K^+^ at low potentials, which restricted the vibration of the A_1g_ vibrational mode dominated by *sp*^2^ hybridization and thus reduced degree of defect. However, the vibrational mode (*E*_2g_) of short-range ordered graphitic domain structure near the large mesoporous pores could be obtained by intercalation chemistry (Fig. 7e) [67]. Therefore, the NSLPC-700 anode with an abundant “defect in disorder” structure was more beneficial for K^+^ adsorption and provided the most excellent potassium storage kinetics.

Based on the above systematic studies, we can attribute the superior potassium storage performance of the NSLPC-700 anode to the following perspectives: (i) NSLPC-700 has the highest nitrogen doping level of 21.6 at%, forming a “defect in disorder” structure, which provides abundant active sites for K^+^ and thus improves potassium storage kinetics; (ii) The appropriate SSAs and rich mesoporous structure of NSLPC-700 provides an efficient diffusion pathway for K^+^, while the nanosheet structure of NSLPC-700 shortens the K^+^ diffusion distance and thus improves the potassium-ion storage kinetics. (iii) Based on the theoretical calculations, the co-doping of N/S cannot effectively enhance the adsorption energy of K^+^ compared with nitrogen doping only, but can improve the electrochemical performance of potassium storage by improving the wettability between the electrode and the electrolyte.

**Fig. 8:**
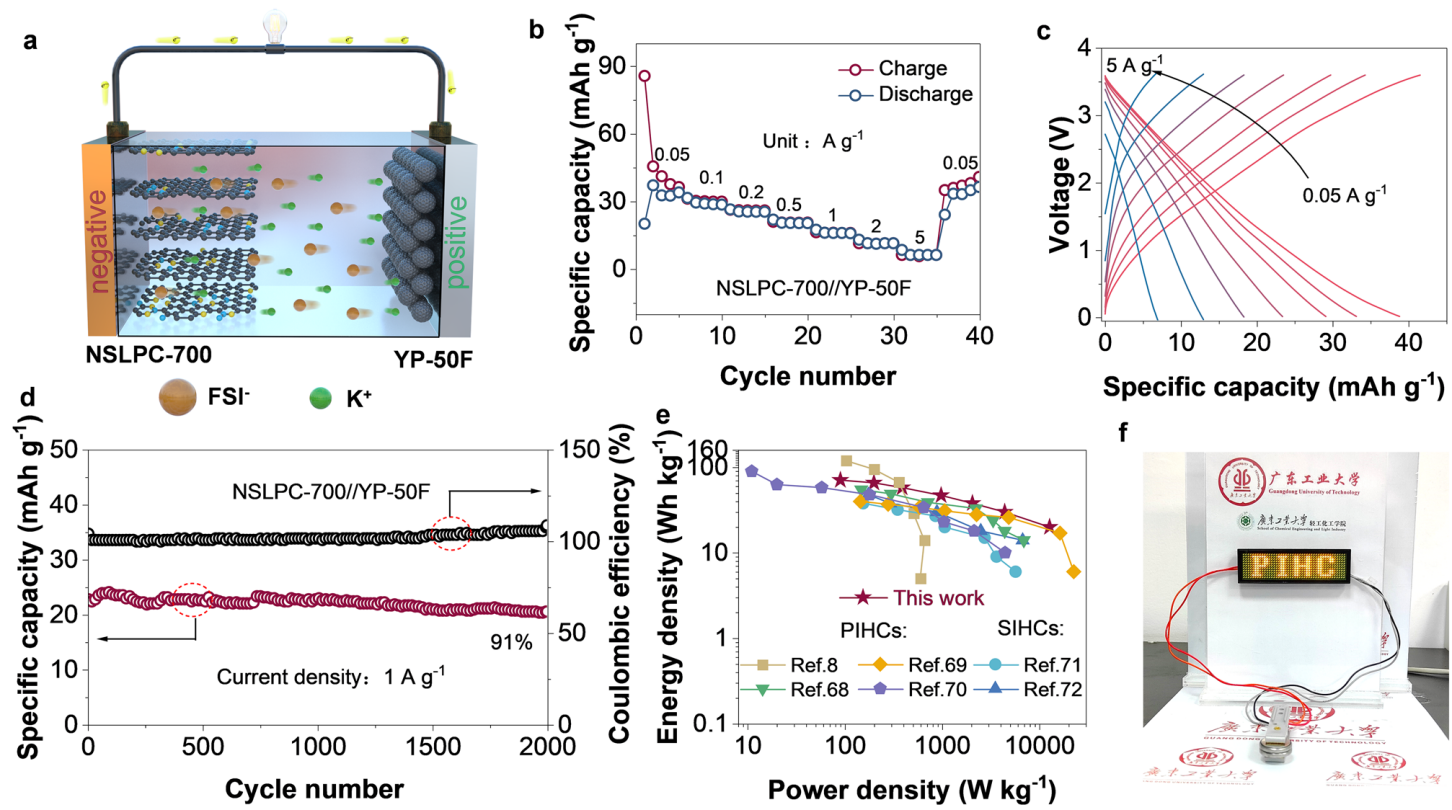
The performance of PIHC. **a** Schematic illustrating the configuration of the PIHC (NSLPC-700//YP-50F) device. **b** Rate performance, and **c** GCD curves at different current densities. **d** Long-term cycle performance at 1 A g^−1^. **e** Ragone curves of the NSLPC-700//YP-50F PIHC compared with other PIHC reported recently. **f** A digital photograph showing an LED bulb is powered by the NSLPC-700//YP-50F PIHC

We assembled a potassium-ion hybrid capacitor (PIHC) using pre-activated NSLPC-700 and commercial activated carbon (YP-50F) as the anode and cathode, respectively, with 3 M KFSI in DME as the electrolyte (NSLPC-700//YP-50F) (Fig. 8a). As shown in Fig. S33a, CV tests for the anode and cathode electrodes were carried out in the voltage window of 0.01–3 V and 1.5–3.6 V, respectively [73]. The CV curves of the assembled PIHC in the voltage window of 0.01–3.6 V are displayed in Fig. S34. As can be seen from Fig. S34, its CV curve exhibits a rectangular-like shape (1 mV s^–1^), indicating a typical excellent electrochemical capacitor behavior. Meanwhile, the YP-50F cathode exhibits excellent rate performance (45 mAh g^–1^ specific capacity at 0.1 A g^–1^) (Fig. S33b). Notably, the assembled PIHC possesses a reversible specific capacity (Fig. 8b) of 39 mAh g^–1^ at a current density of 0.05 A g^–1^ (calculated from the total mass of the anode and cathode). Meanwhile, the GCD curves indicate excellent electrochemical capacitor behavior (Fig. 8c). The PIHC still has a Coulombic efficiency of 100% and a capacity retention of 91% (Fig. 8d) after 2000 cycles at 1 A g^–1^. Moreover, it maintains a capacity retention rate of 89% at 0.2 A g^–1^ over 100 cycles (Fig. S35). The high reversible specific capacity and excellent cycling stability of the PIHC originate from “defect in disorder” structure in the NSLPC-700, which can efficiently adsorb large amounts of K^+^ and thus improve potassium-ion storage performance. Notably, the PIHC delivers a high energy density of 71 Wh kg^–1^ (91 W kg^–1^) and a high power density of 12,875 W kg^–1^ (Fig. 8e). Meanwhile, it is also superior to other PIHC and sodium ion hybrid capacitors (SIHCs) (Fig. 8e). To illustrate the potential of PIHC for application, a 3 V LED lamp was powered by connecting two-coin cells in series (Fig. 8f). Thus, we prepared NSLPC-700 porous carbon, which shows great potential for practical applications. In summary, the NSLPCs with a “defect in disorder” structure provide a new idea for the preparation of high-performance carbon anode materials for electrochemical energy storage devices.

## Conclusion

Highly heteroatom-doped carbon anodes with excellent electrochemical performance were prepared using a new supermolecule-mediated strategy from general biomass precursors. Based on this novel method, we achieved a carbonaceous material with 21.6 at% of ultra-high nitrogen atom doping (18.3 at% edge nitrogen doping). The ultra-high nitrogen doping originates from the cleavage of the intermediate C_3_N_4_ product covalently bonded within the carbon skeleton. Due to the well-dispersed state of lignin by constructing supermolecules, the obtained carbon showed nanosheet morphologies. Notably, this carbon anode showed a high content of edge defects and a unique structure exhibiting remarkable potassium-ion storage performance. This general strategy could be developed to synthesize other highly doped carbons from general biomasses.

### Supplementary Information

Below is the link to the electronic supplementary material.

Supplementary file1 (PDF 2932 KB)
