# Supplementary material for: Defect Engineering of Disordered Carbon Anodes with Ultra-High Heteroatom Doping Through a Supermolecule-Mediated Strategy for Potassium-Ion Hybrid Capacitors
Source: Nanomicro Lett. 2023 Jan 27;15:41. doi: 10.1007/s40820-022-01006-0 (PMC9883381; doi:10.1007/s40820-022-01006-0)
Supplement: Supplementary file 1 — Supplementary file1 (PDF 2932 KB) [file 40820_2022_1006_MOESM1_ESM.pdf]

Supporting Information for

Defect Engineering of Disordered Carbon Anodes with Ultra-High Heteroatom Doping through A Supermolecule-Mediated Strategy for Potassium Ion Hybrid Capacitors

Lei Zhao[1, a], Shirong Sun[1, a], Jinxin Lin[1, a], Lei Zhong[1], Liheng Chen[1], Jing Guo[3], Jian Yin[4], Husam N. Alshareef[4, *], Xueqing Qiu[1, 2, *] and Wenli Zhang[1, 2, 5, *]

[1] Guangdong Provincial Key Laboratory of Plant Resources Biorefinery, School of Chemical Engineering and Light Industry, Guangdong University of Technology (GDUT), 100 Waihuan Xi Road, Panyu District, Guangzhou 510006, P. R. China

[2] Jieyang Branch of Chemistry and Chemical Engineering Guangdong Laboratory (Rongjiang Laboratory), Jieyang 515200, P. R. China

[3] Key Laboratory of Dielectric and Electrolyte Functional Material Hebei Province, School of Resources and Materials, Northeastern University at Qinhuangdao, Qinhuangdao 066004, P. R. China

[4] Materials Science and Engineering, Physical Science and Engineering Division, King Abdullah University of Science and Technology (KAUST), Thuwal 23955-6900, Saudi Arabia

[5] Research Institute of Green Chemical Engineering and Advanced Materials, School of Advanced Manufacturing, Guangdong University of Technology (GDUT), Jieyang, Jieyang 515200, P. R. China

[a] Lei Zhao, Shirong Sun, and Jinxin Lin contributed equally to this work.

* Corresponding authors. E-mail: husam.alshareef@kaust.edu.sa (H. N. Alshareef) cexqqiu@scut.edu.cn (X. Qiu); wlzhang@gdut.edu.cn (W. Zhang)

S1 Experimental Methods

S1.1 Electrochemical Equations and Calculations

In PIHCs full-cell tests, the calculations of energy (*E*, $Wh\ kg^{-1}$) and power densities (*P*, $W\ kg^{-1}$) based on the total mass of both anode and cathode materials were performed using the equations below [S1, S2]:

$$P = \Delta V \times i \tag{S1}$$

$$E = P \times t/3600 \tag{S2}$$

$$\Delta V = (V_{max} + V_{min})/2 \tag{S3}$$

in which t (s) is the discharge time, *i* (A g^{-1}) is the charge/discharge current, V_{max} (V) is the discharge potential excluding the IR drop, and V_{min} (V) is the potential at the end of discharge voltages.

The contributions to capacitive controlled and diffusion-controlled processes are estimated as follows [S3]:

$$i = av^b \tag{S4}$$

$$i = k_1 v + k_2 v^{\frac{1}{2}} \tag{S5}$$

or

$$\frac{i}{v^{\frac{1}{2}}} = k_1 v^{\frac{1}{2}} + k_2 \tag{S6}$$

where i is the instantaneous current density, v is the scanning rate, and a and b are adjustable parameters obtained from the v_1 and v_2 scanning rates, respectively. The electrode reaction is controlled by the diffusion process when the value of b is 0.5, and the electrode response is controlled by the surface-dominated process when the b value is 1. The contributions of the capacitive current and the diffusion-controlled current to the whole electrode reaction process are represented by the k_1v and $k_2v^{1/2}$ in equations (S5) and (S6), respectively.

The diffusion coefficients of K^+ were calculated from the Galvanostatic Intermittent Titration Technique (GITT) using the following equation [S4].

$$D_{K^+} = \frac{4}{\pi\tau}\left(\frac{m_B V_M}{M_B S}\right)^2\left(\frac{\Delta E_S}{\Delta E_\tau}\right)^2 \quad \text{(S7)}$$

Where τ denotes the duration of the current pulse, s; m_B is the mass of electrode active material, g; S is the geometric area of the electrode, cm^2; ΔE_s is the quasi-thermodynamic equilibrium potential difference before and after the current pulse, V; ΔE_t is the potential difference during current pulse neglecting the IR-drop, V; V_M is the molar volume of the active materials, M_B is the molar mass of carbon. The value of M_B/V_M is the density of electrodes (1.5 g cm^{-3}), which is estimated to be lower than the density of graphite (2.2 g cm^{-3}). Before the GITT measurement, the NSLPCs electrodes were pre-cycled for three cycles under a constant current density of 50 mA g^{-1}.

S2 Molecular Dynamic Simulation and Density Functional Theory Calculation

Firstly, Hundreds of initial configurations were generated by the genmer module of the Molclus program [S5]. All the clusters were then pre-optimized under the semi-empirical method GFN2-Xtb [S6]. The obtained configurations with the low energy were further optimized and calculated the frequency under B3LYP-D3(BJ)/6-31G* level using Gaussian16 package with the consideration of implicit solvent model. The interaction energy between the LS and MA+TCA and the energy among the MA and TCA molecules were calculated with the correction of basis set superposition error (BSSE) under the B3LYP-D3(BJ)/6-311G** level. To study the weak interaction among the molecules, the Independent gradient model based on Hirshfeld partition (IGMH) [S7] were analyzed by Multiwfn 3.8 (dev) program [S8] and visualized by VMD 1.9.4. software [S9].

All the calculations are performed in the framework of the density functional theory with the projector augmented plane-wave method, as implemented in the Vienna ab initio simulation package [S10]. The generalized gradient approximation proposed by Perdew, Burke, and Ernzerhof is selected for the exchange-correlation potential [S11]. The long-range van der Waals interaction is described by the DFT-D3 approach [S12]. The cut-off energy for the plane wave is set to 500 eV. The energy criterion is set to 10-6 eV in the iterative solution of the Kohn-Sham equation. The Brillouin zone integration is performed at the Gamma point with a $2 \times 2 \times 1$ k-mesh grid for structural optimization calculations and a $6\times6\times1$ k-mesh grid for the electronic structure calculations. All the structures are relaxed until the residual forces on the atoms have declined to less than 0.03 eV Å^{-1}. The vacuum thickness along the z-axis is set to 15 Å, which is large enough to avoid interaction between periodic images.

S3 Supporting Figures and Tables

Table S1 Porosity parameters of NSLPCs samples

Sample	S_{BET} (m^2 g^{-1})	V_{Total} (cm^3 g^{-1})	V_{micro} (cm^3 g^{-1})	V_{meso} (cm^3 g^{-1})	V_{micro}/V_{total} (%)	D_{ave} (nm)
NSLPC-700	229	0.56	0.02	0.54	96.4	10.8
NSLPC-800	362	0.95	0.06	0.89	93.6	18.1
NSLPC-900	322	1.52	0.02	1.50	98.7	21.5
NSLPC-1000	589	1.86	0.08	1.78	95.7	17.3

Table S2 Atomic elemental content of NSLPCs

sample	C (at%)	N (at%)	O (at%)	S (at%)
NSLPC-700	70.0	21.6	7.6	0.8
NSLPC-800	76.4	17.0	6.0	0.6
NSLPC-900	88.2	7.2	4.0	0.6
NSLPC-1000	92.5	2.4	3.9	1.2

Table S3 Table of binding energies and element content for different bond types

	Bonding type	NSLPC-700	NSLPC-800	NSLPC-900	NSLPC-1000
C 1s	C=C/C-C	284.8	284.8	284.8	284.8
	C-N	286.0	285.8	285.8	285.9
	C-S	287.2	287.9	287.3	287.0
	C=O	288.7	289.7	229.5	288.8
	O-C=O	291.0	291.8	291.6	290.7
O 1s	C=O	531.4	530.9	530.9	531.0
	C-OH/C-O-C	532.5	532.2	532.1	532.4
	-COOH/H_2O	534.0	533.4	533.2	533.5
N 1s	Pyridinic-N	398.2	398.2	398.1	398.3
	Pyrrolic-N	399.6	399.7	399.6	399.2
	Graphitic-N	400.2	400.3	400.8	401.2
	Oxidized-N	401.2	402.4	402.6	403.2
S 2p	S $2p_{3/2}$ (C-S-C)	163.9	163.7	163.8	163.7
	S $2p_{1/2}$ (C-SO_X-C)	165.1	165.2	164.7	164.9
	Oxidized-S (-SO_X-)	167.4/168.5	167.5/168.8	167.3/168.6	167.5/168.7

Table S4 Different chemical bonding concentrations of NSLPCs

	Bonding type	NSLPC-700 (%)	NSLPC-800 (%)	NSLPC-900 (%)	NSLPC-1000 (%)
C 1s	C=C/C-C	51.3	39.5	55.8	61.8
	C-N	27.3	46.2	24.1	16.8
	C-S	9.6	6.7	10.2	10.1
	C=O	7.7	5.6	7.4	5.0
	O-C=O	4.1	1.9	2.6	6.3
O 1s	C=O	44.8	39.0	51.1	51.1
	C-OH/C-O-C	37.2	26.9	23.8	28.2
	-COOH/H_2O	18.0	34.1	25.1	20.6
N 1s	Pyridinic-N	**42.7**	35.7	31.2	13.8
	Pyrrolic-N	**42.3**	45.8	18.2	14.8
	Graphitic-N	8.6	8.3	27.1	44.8
	Oxidized-N	6.4	10.2	22.9	26.7
S 2p	S $2p_{3/2}$ (C-S-C)	26.9	12.4	29.1	34.7
	S $2p_{1/2}$ (C-SO_X-C)	26.4	49.7	13.4	37.2
	Oxidized-S (-SO_X-)	43.3	37.9	57.5	28.1

Table S5 Comparison of different defective carbon anode materials of PIHCs

Materials	Current density (A g^{-1})	Initial coulombic Efficiency (%)	Refs.
S/N@C	0.05	24.6	Adv.Mater.2019, 31, 1805430 DOI:10.1002/adfm.201801989
nitrogen-doped graphitic nanocarbons-600	0.05	15.5	Adv. Funct. Mater. 2019, DOI: 10.1002/adfm.2019036411903641.
nitrogen-doped hierarchical porous hollow carbon spheres	0.028	37.1	Adv. Funct. Mater. 2019, DOI: 10.1002/adfm.2019034961903496.
nitrogen/oxygen in situ dual-doped hierarchical porous hard carbon	0.05	25	Adv. Mater. 2018, 30, 1700104. DOI:10.1002/adma.201700104
N/O dual-doped carbon network	0.05	47.1	Energy Storage Mater. 2019, DOI: 10.1016/j.ensm.2019.05.037
three-dimensional nitrogen-doped framework carbon	0.1	24.3	Energy Storage Mater. 2019, DOI: 10.1016/j.ensm.2019.04.008.

carbon nanosheets750	0.05	38	Adv. Energy Mater. 2019, 9, 1803894. DOI:10.1002/aenm.201803894
nitrogen-doped carbon nanosheets	0.05	20	Adv. Funct. Mater. 2018, 28, 1801989. DOI:10.1002/adfm.201801989
**NSLPC-700**	**0.05**	**48.0**	**This work**

Table S6 Comparison of different defective carbon anode materials for PIHCs

Anode	Specific capacity	Rate capability	Cyclic stability	Refs.
N-doped hollow carbon	307 mAh g^{-1} at 0.05 A g^{-1}	200 mAh g^{-1} at 1 A g^{-1}	160 mAh g^{-1} after 800 cycles at 1 A g^{-1}	Nano Energy 2019, 65, 104038.
N, S co-doped carbon	276 mAh g^{-1} at 0.1 A g^{-1}	218.5 mAh g^{-1} at 3 A g^{-1}	144.9 mAh g^{-1} after 1200 cycles at 3 A g^{-1}	Adv. Energy Mater. 2019, 9, 1901379.
N, S co-doped 3D porous carbon nanosheets	392 mAh g^{-1} at 0.1 A g^{-1}	265 mAh g^{-1} at 1 A g^{-1}	271 mAh g^{-1} after 1000 cycles at 1 A g^{-1}	Adv.Energy Mater. 2019, 9, 1901533
Pitch-derived soft carbon	296 mAh g^{-1} at 0.1 C	115.2 mAh g^{-1} at 5 C	retention after 1000 cycles at 1 C	Adv.Mater. 2020, 32, 2000505.
N/O dual-doped hard carbon	304.6 mAh g^{-1} at 0.1 A g^{-1}	223.4 mAh g^{-1} at 1 A g^{-1}	189.5 mAh g^{-1}after 5000 cycles at 1 A g^{-1}	Adv. Sci. 2020, 7, 1902547.
Highly N-doped carbon nanofibers	217 mAh g^{-1} at 0.2 A g^{-1}	101 mAh g^{-1} at 20 A g^{-1}	191 mAh g^{-1}after 200 cycles at 0.2 A g^{-1}	Nat. Commun. 2018, 9, 1720.
Edge N-doped carbon spheres	290 mAh g^{-1} at 0.2 A g^{-1}	195 mAh g^{-1} at 1 A g^{-1}	305 mAh g^{-1} after 600 cycles at 0.2 A g^{-1}	Angew. Chem. Int. Ed. 2020, 59, 4448.
3D Carbon framework	348.2 mAh g^{-1} at 0.05 A g^{-1}	245 mAh g^{-1} at 2 A g^{-1}	188.8 mAh g^{-1} after 2000 cycles at 1 A g^{-1}	ACS Energy Lett. 2020, 5, 1653.
**NSLPC-700**	**419 mAh g^{-1} at 0.05 A g^{-1}**	**246 mAh g^{-1} at 1 A g^{-1}**	**179 mAh g^{-1} after 1500 cycles at 2 A g^{-1}**	**This work**

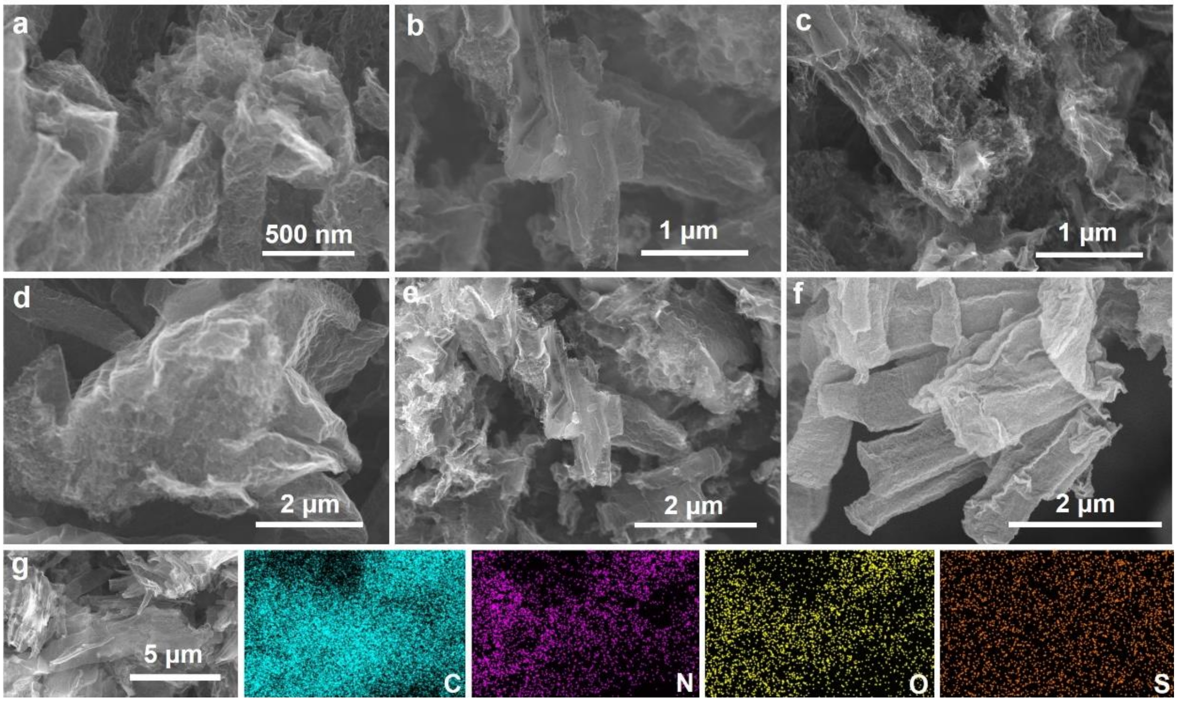

Fig. S1 (**a, d**) SEM image of NSLPC-800, (**b, e**) SEM image of NSLPC-900, (**c, f**) SEM image of NSLPC-1000, and the (**g**) Energy dispersive spectroscopy (EDS) mapping images of NSLPC-800 for the elements of carbon, nitrogen, oxygen, and sulfur

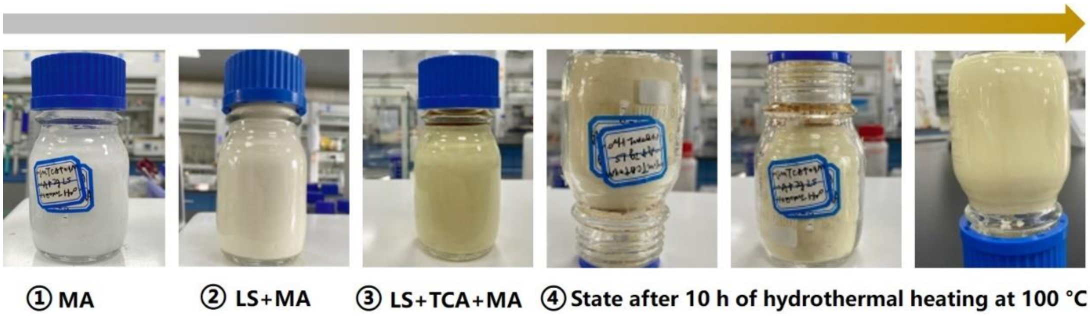

Fig. S2 Digital figures for the different states for the synthesis of the supermolecule precursors at each stage

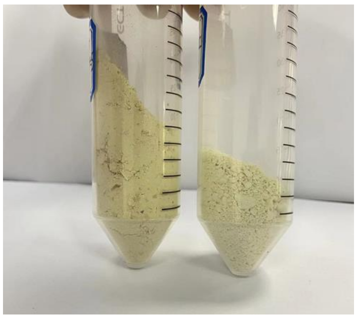

Fig. S3 Digital pictures of a mixture of (left) TCA+MA+LS and a mixture of (right) TCA+MA

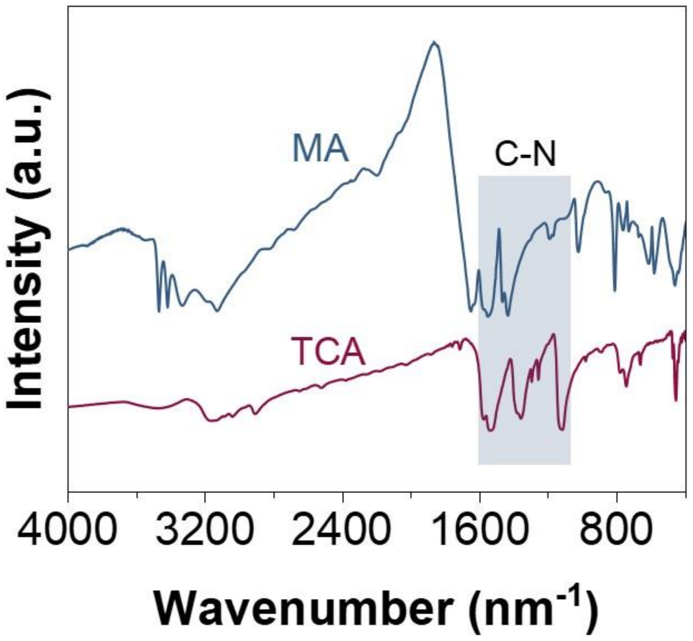

Fig. S4 FT-IR spectra of the TCA and MA

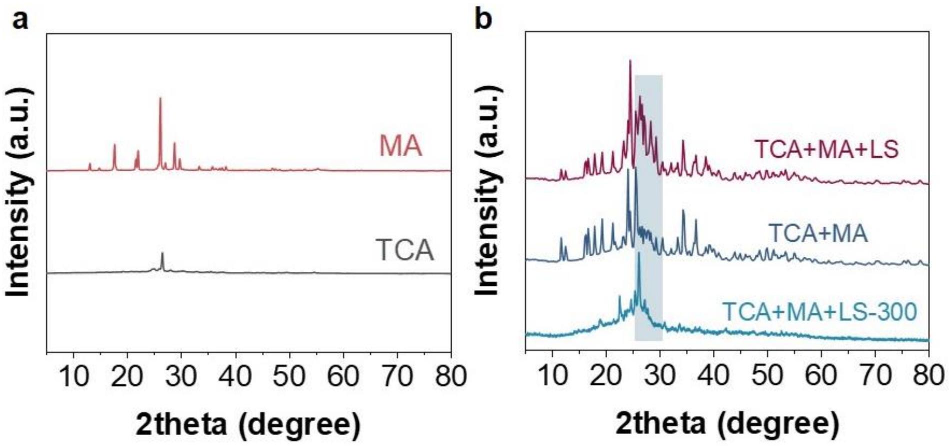

Fig. S5 (**a**) The XRD patterns of TCA and MA, (**b**) The XRD patterns of TCA+MA+LS, TCA+MA, and TCA+MA+LS-300 (heat-treated at 300 ^{o}C). Heat treatment of TCA+MA+LS would not cause apparent change in its XRD patterns

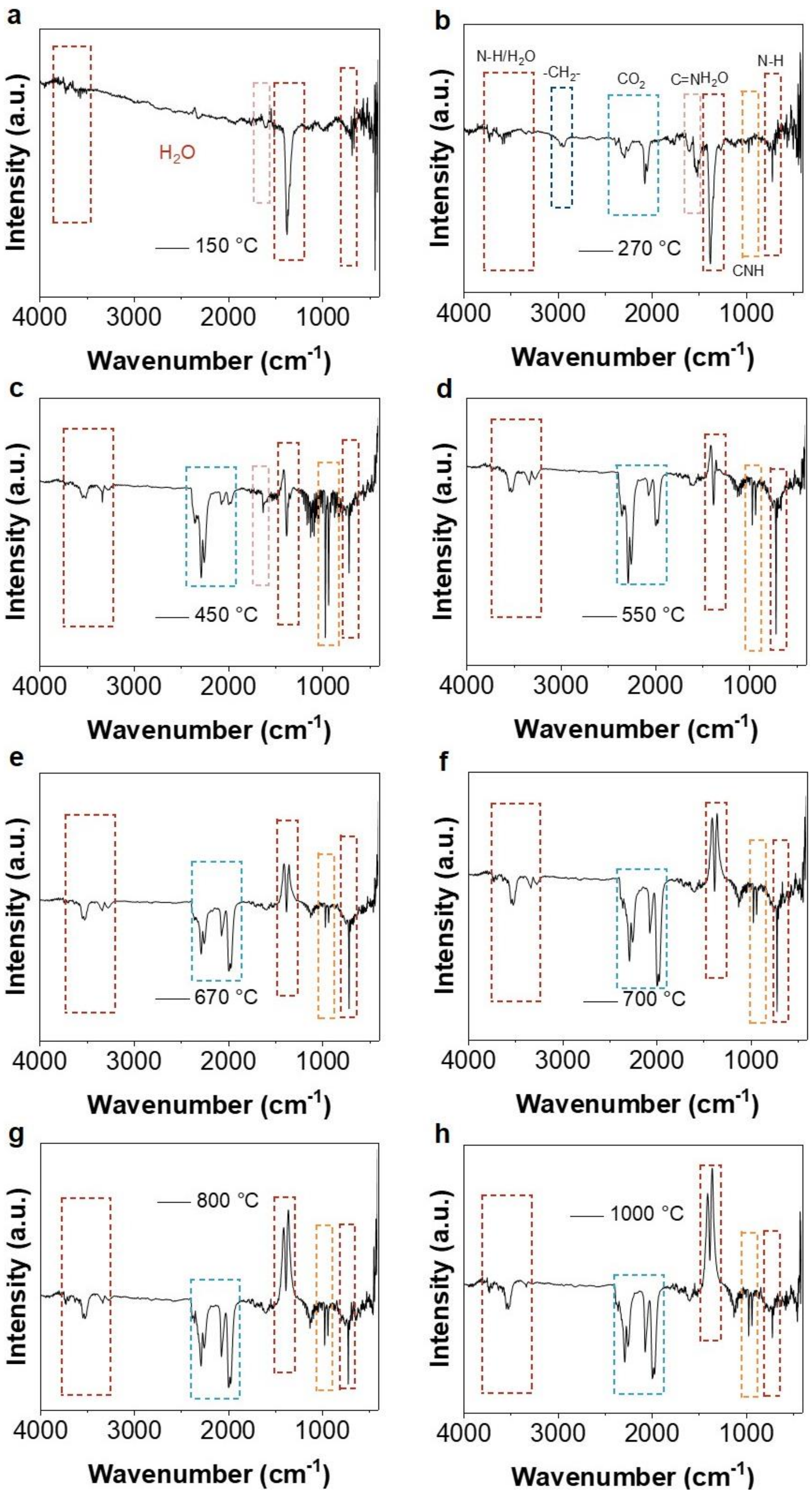

Fig. S6 FT-IR spectra of the pyrolysis product of TCA+MA+LS precursors at different temperatures

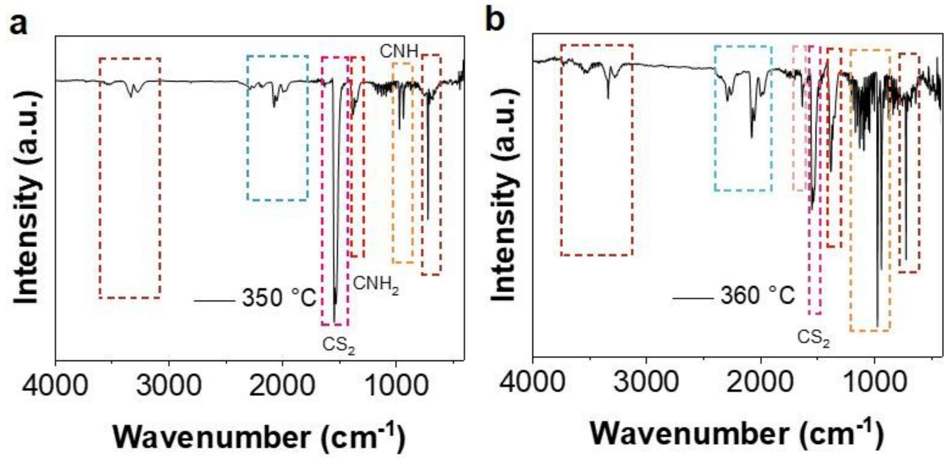

Fig. S7 (**a, b**) FT-IR spectra of TCA+MA+LS precursors heat-treated at temperatures of 350 ℃ and 360 ℃

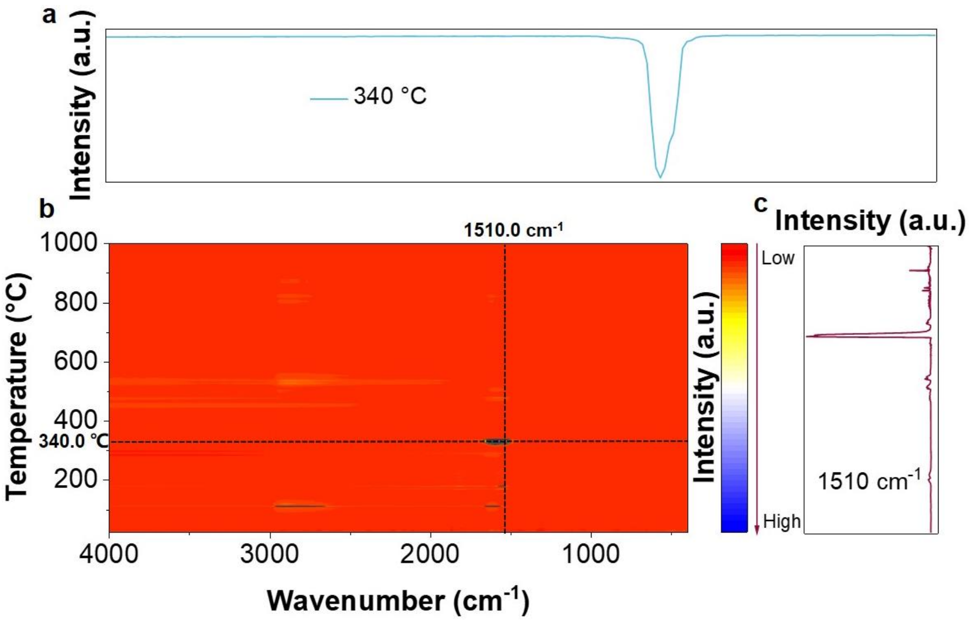

Fig. S8 2D temperature-dependent FT-IR spectrum of TCA+MA+LS precursor

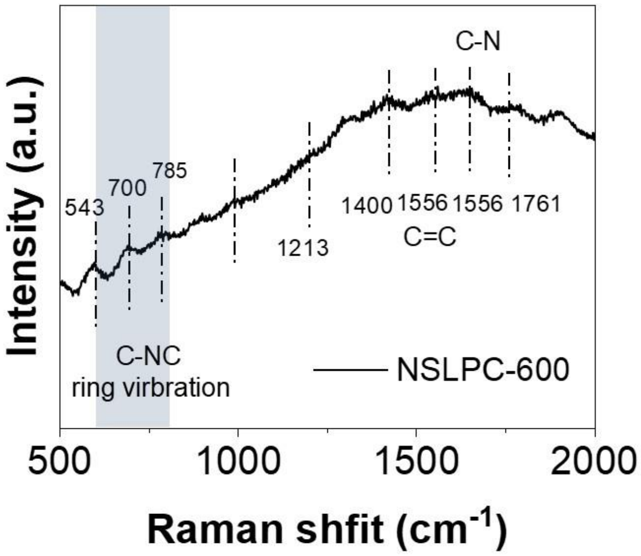

Fig. S9 Raman spectra of the NSLPC-600

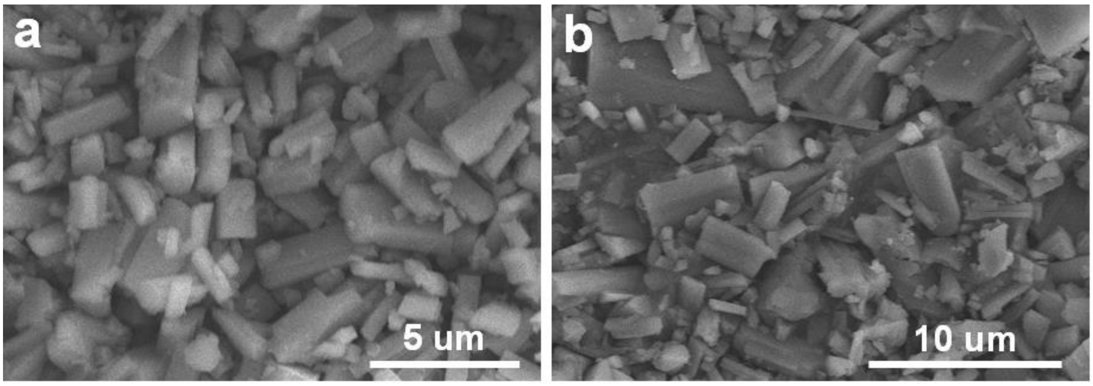

Fig. S10 SEM images of the assembly of (**a**) TCA+MA and (**b**) TCA+MA+LS

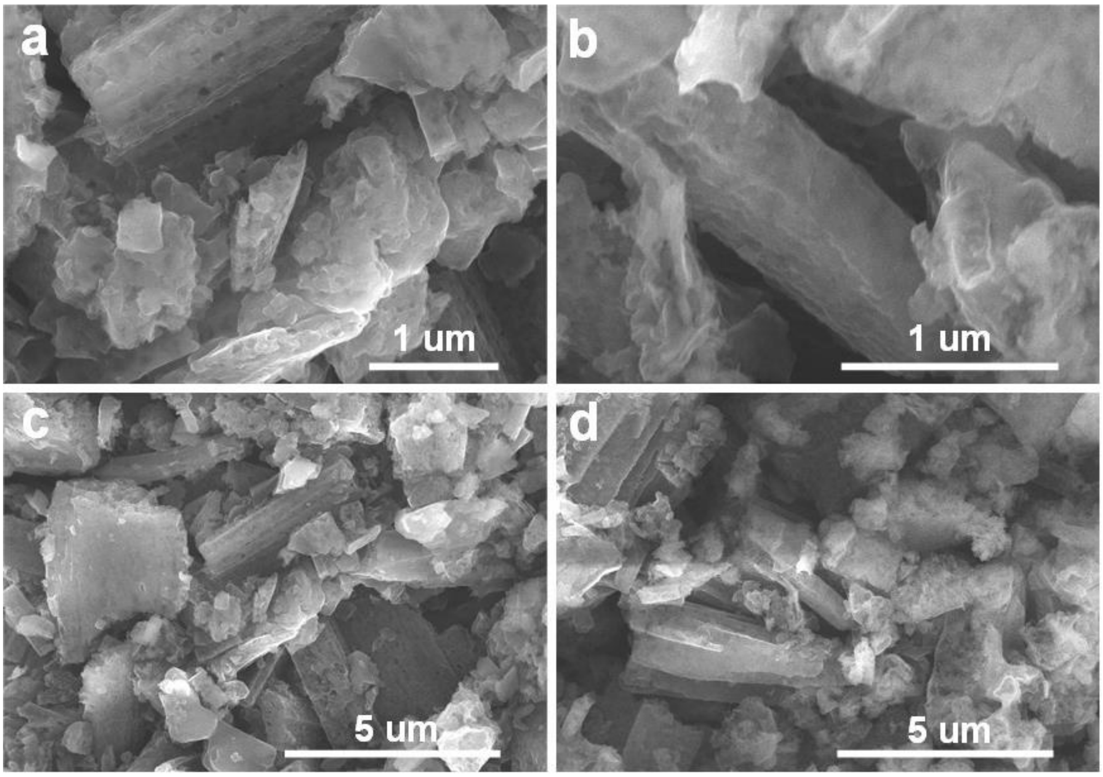

Fig. S11 SEM images of (**a, c**) NSLPC-550 and (**b, d**) NSLPC-600

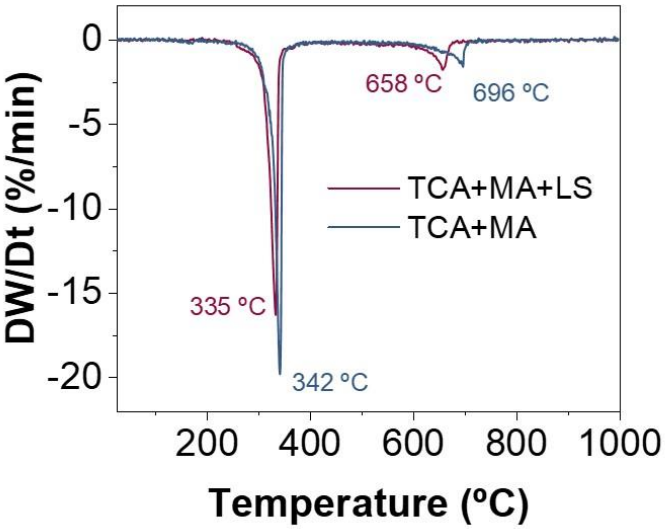

Fig. S12 The differential TGA curves of TCA+MA+LS and TCA+MA

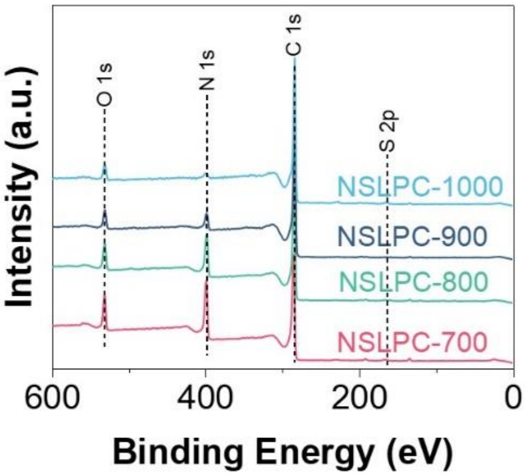

Fig. S13 XPS spectra of NSLPC-700, NSLPC-800, NSLPC-900, and NSLPC-1000

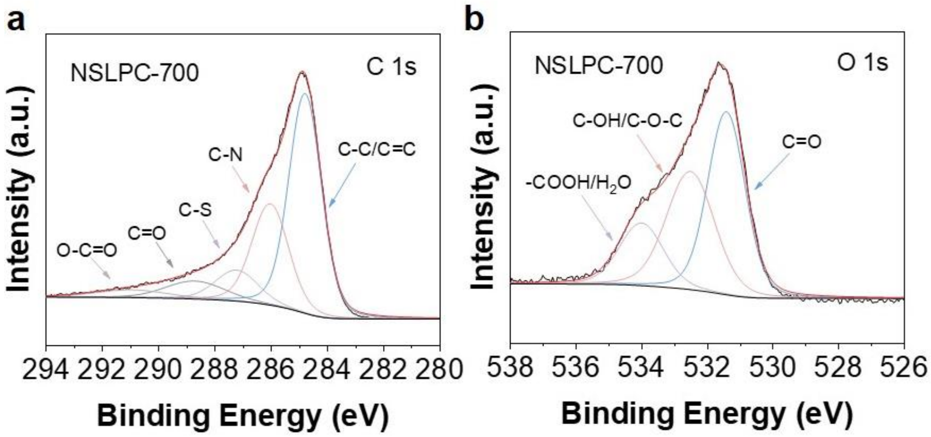

Fig. S14 High-resolution (**a**) C 1s and (**b**) O 1s XPS spectra of NSLPC-700

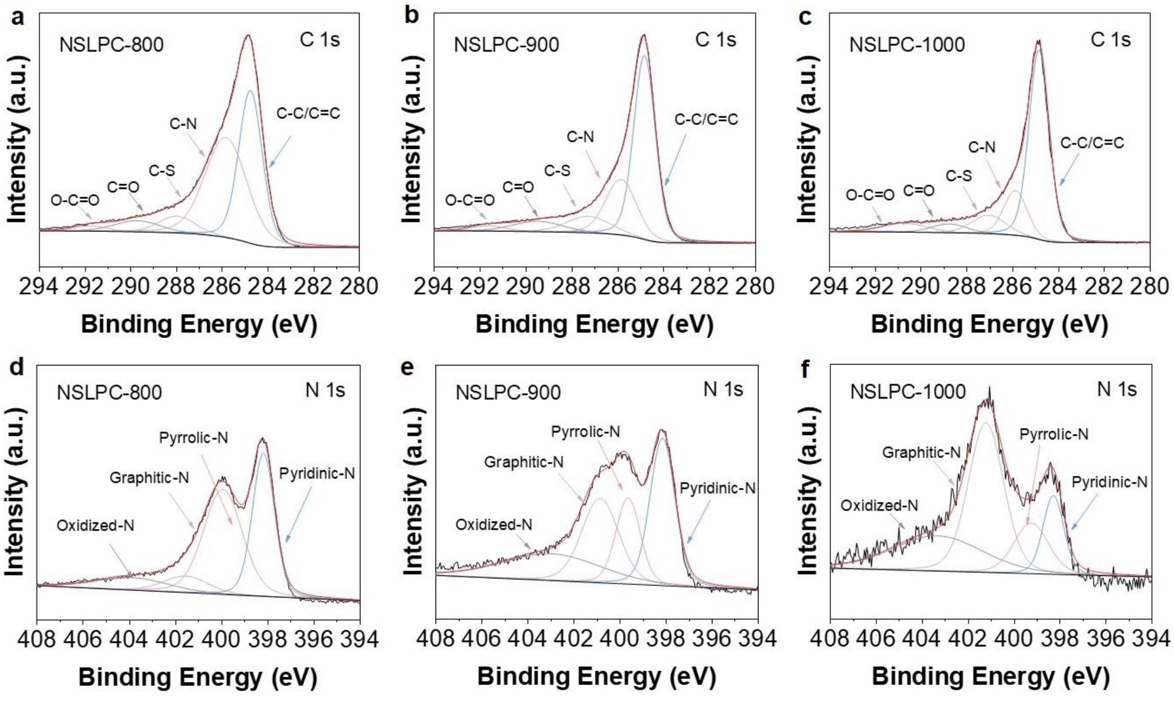

Fig. S15 High-resolution (**a**) C 1s and (**d**) N 1s XPS spectra of the NSLPC-800. High-resolution (**b**) C 1s and (**e**) N 1s XPS spectra of the NSLPC-900. High-resolution (**c**) C 1s, (**f**) N 1s XPS spectra of the NSLPC-1000

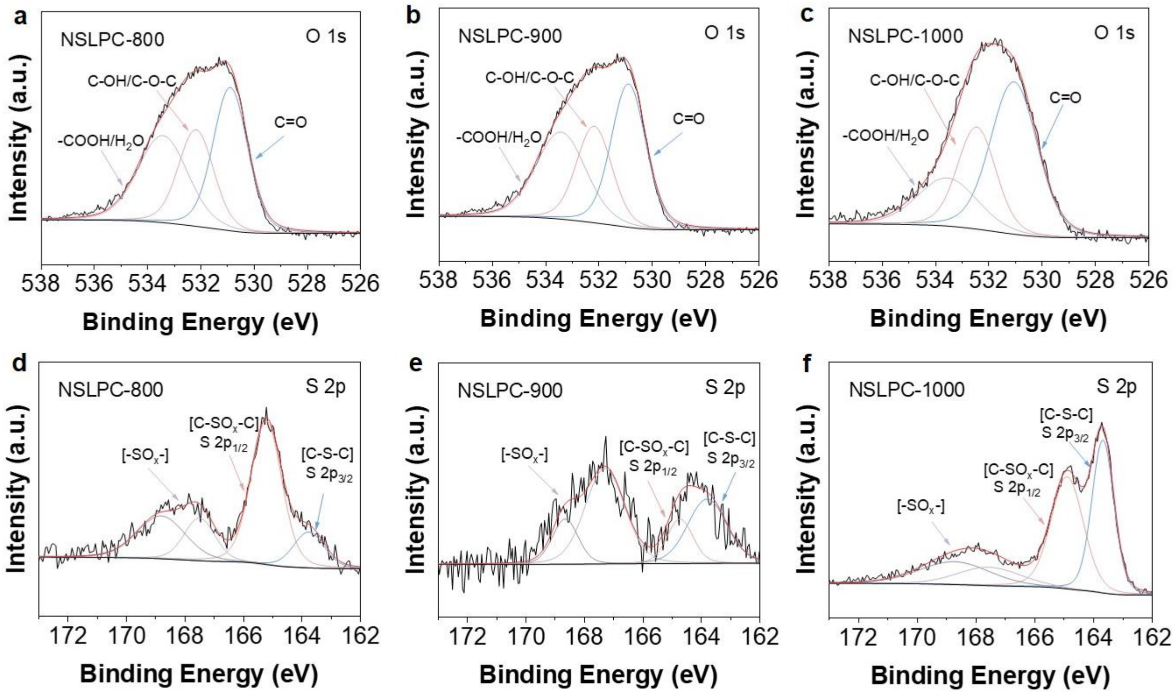

Fig. S16 High-resolution (**a**) O 1s and (**d**) S 2p XPS spectra of the NSLPC-800. High-resolution (**b**) O 1s and (**e**) S 2p XPS spectra of the NSLPC-900. High-resolution (**c**) O 1s and (**f**) S 2p XPS spectra of the NSLPC-1000

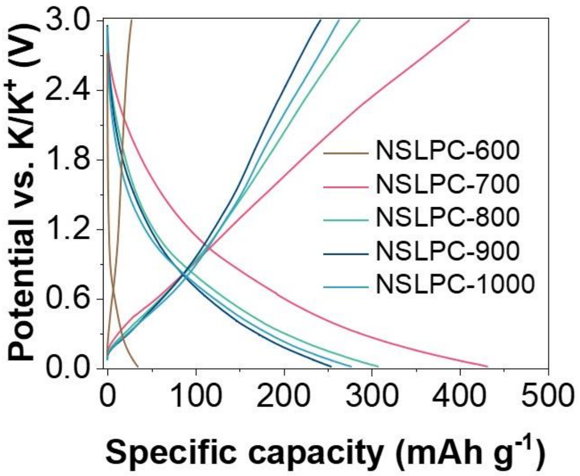

Fig. S17 Comparison of the GCD curves of different samples at 0.05 A g^{-1}

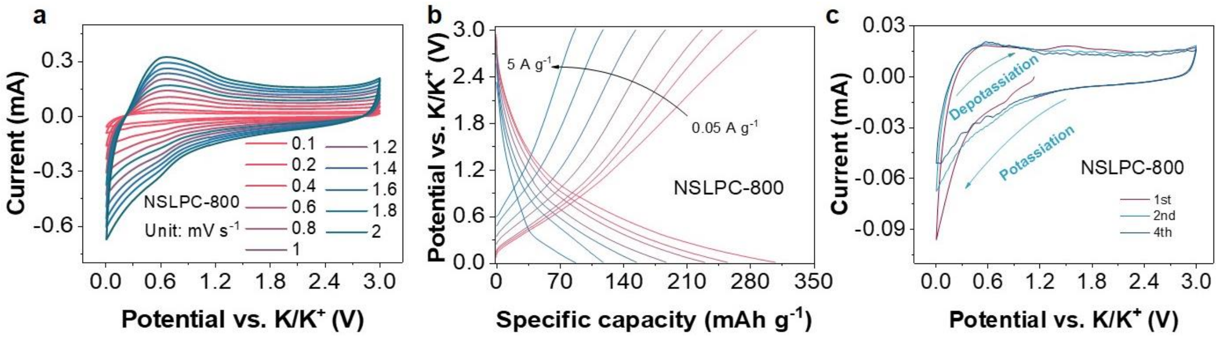

Fig. S18 (**a**) CV curves of NSLPC-800 anode at different scan rates. (**b**) GCD curves of NSLPC-800 anode at different current densities. (**c**) CV curves of NSLPC-800 anode at a scan rate of 0.1 mV s^{-1}

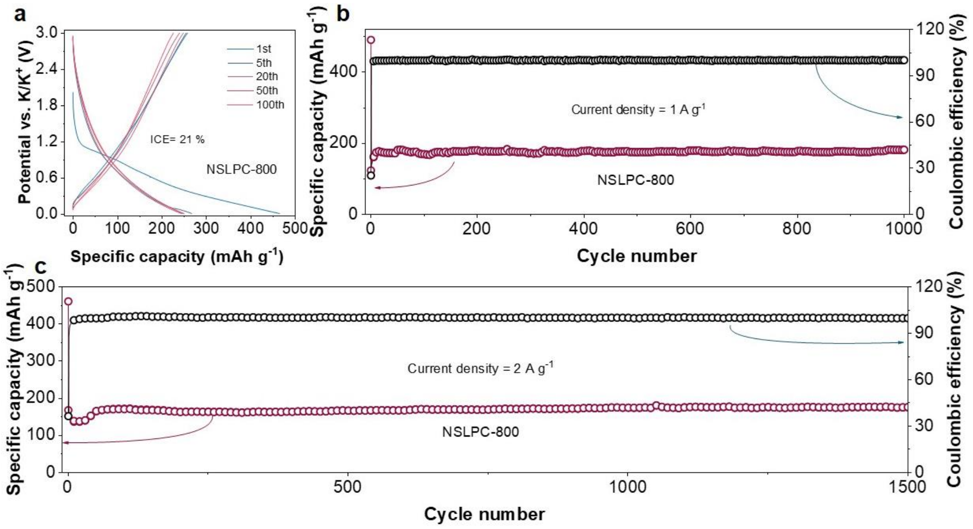

Fig. S19 (**a**) GCD curves of NSLPC-800 anode at a current density of 0.05 A g^{-1}. GCD cycling performance of the NSLPC-800 anode at current densities of (**b**) 1 A g^{-1} and (**c**) 2 A g^{-1}

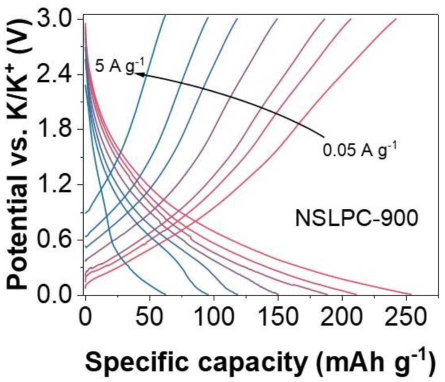

Fig. S20 GCD curves of NSLPC-900 anode at different current densities

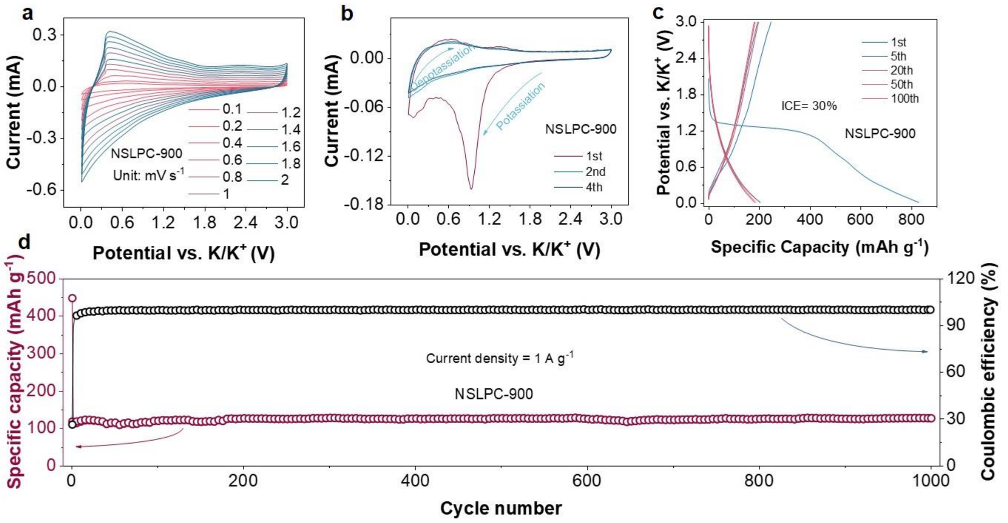

Fig. S21 (**a**) CV curves of NSLPC-900 anode at different scan rates. (**b**) CV curves of NSLPC-900 at initial cycles at a scan rate of 0.1 mV s^{-1}. (**c**) Cycling performance of the NSLPC-900 anode at a current density of 0.05 A g^{-1}. (**d**) Cycling performance of the NSLPC-900 anode at a current density of 1 A g^{-1}

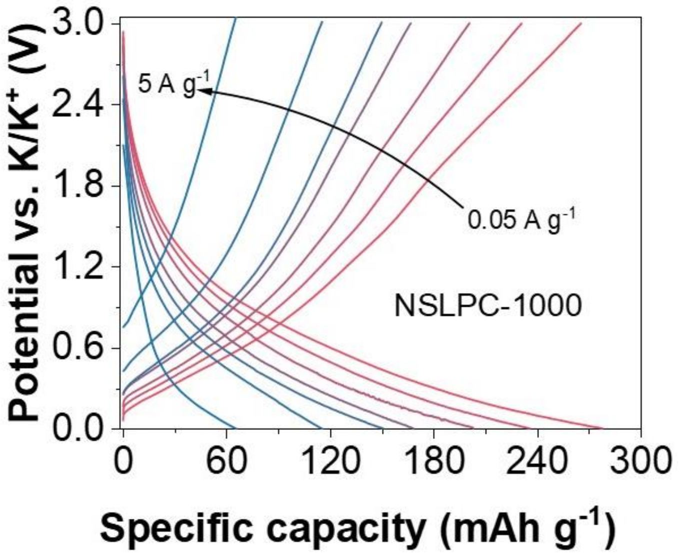

Fig. S22 GCD curves of NSLPC-1000 anode at different current densities

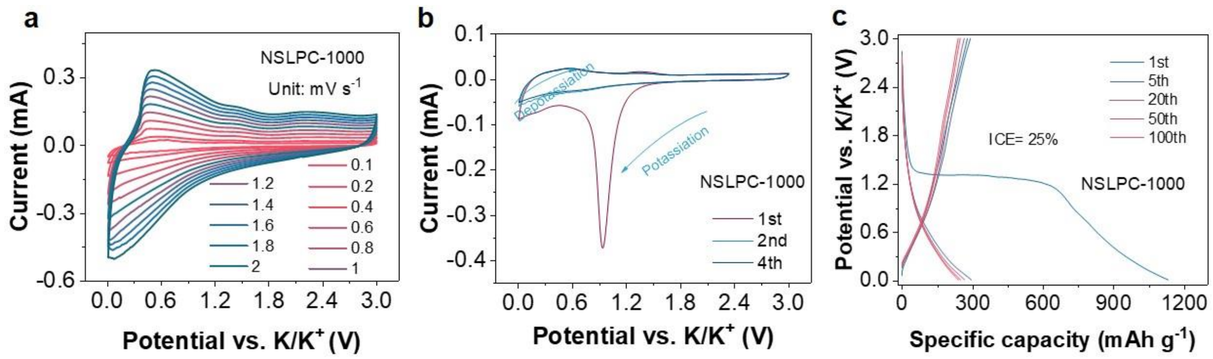

Fig. S23 (**a**) CV curves of NSLPC-1000 at different scan rates. (**b**) CV curves at initial cycles at a scan rate of 0.1 mV s^{-1}. (**c**) GCD curves of NSLPC-1000 anode at a current density of 0.05 A g^{-1}

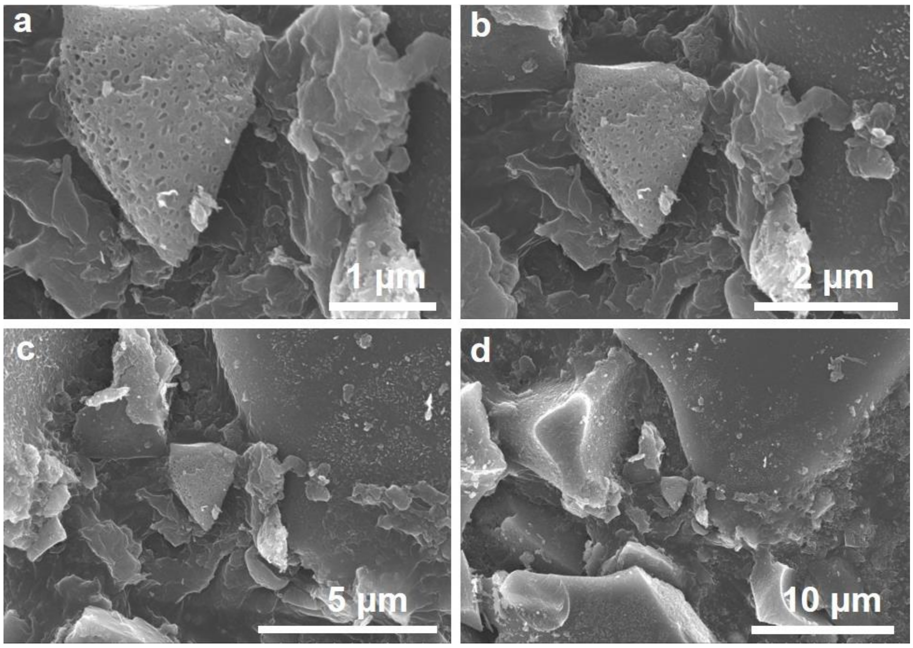

Fig. S24 SEM images of LPC-700

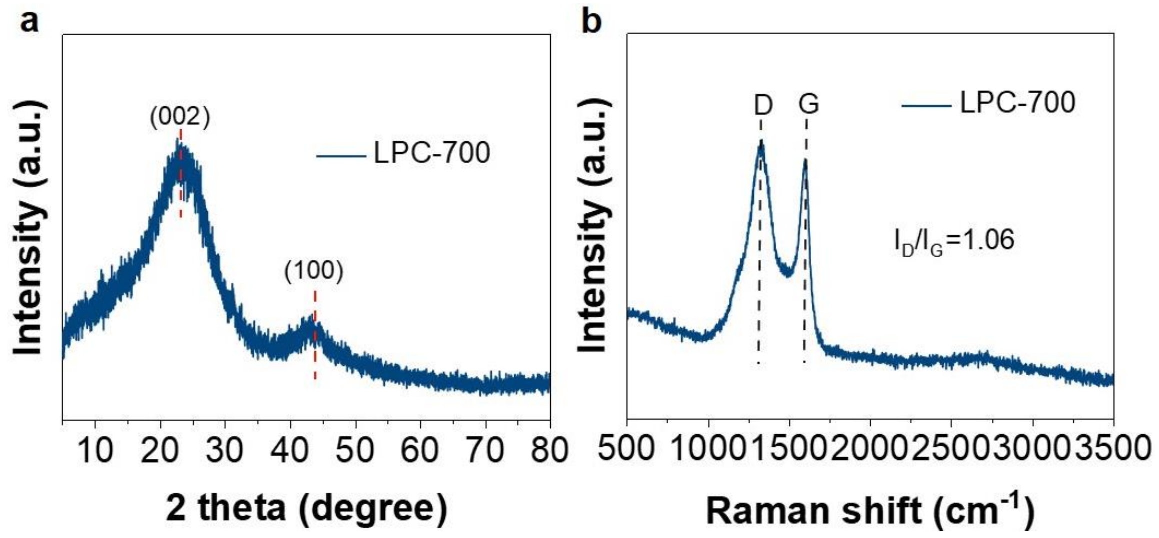

Fig. S25 (**a**) XRD pattern of LPC-700. (**b**) Raman spectra of LPC-700

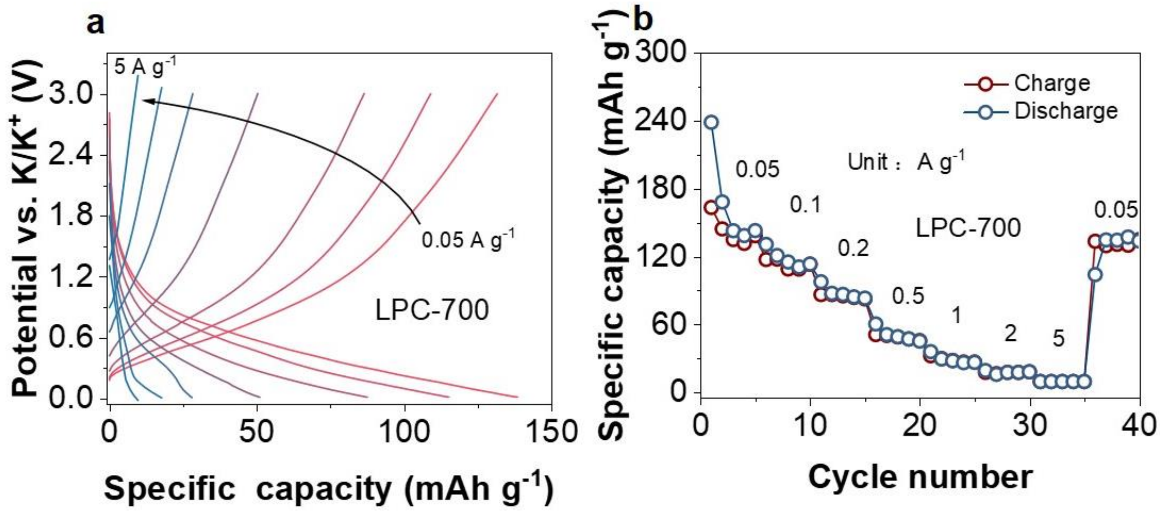

Fig. S26 (**a**) GCD curves of LPC-700 anode at the different current densities. (**b**) Rate performance of LPC-700 anode

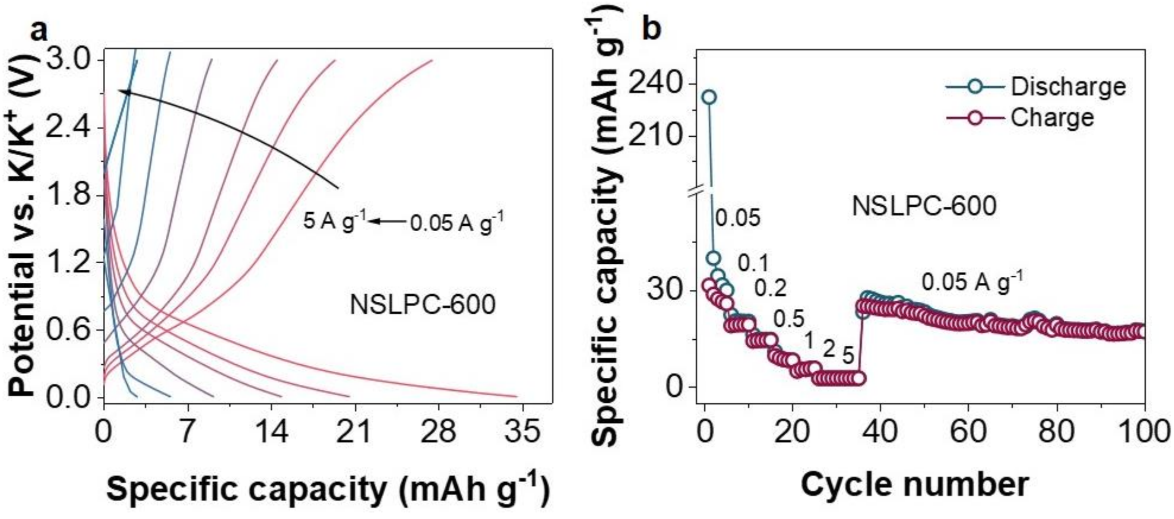

Fig. S27 (**a**) GCD curves of NSLPC-600 anode at different current densities. (**b**) Rate performance of NSLPC-600 anode

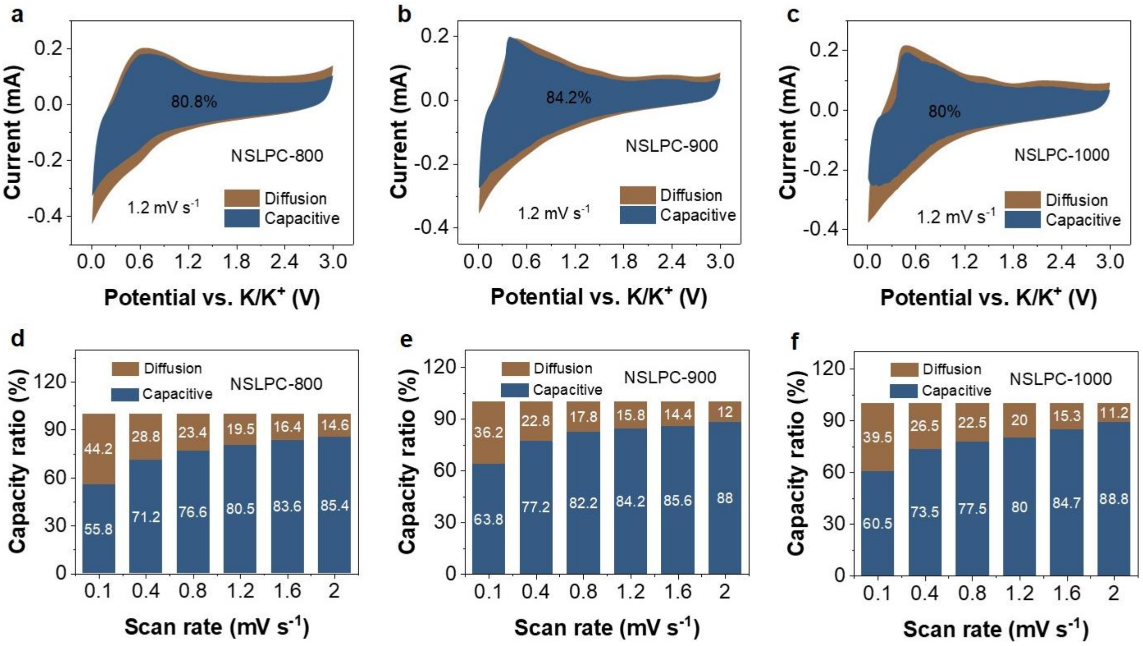

Fig. S28 Capacitive charge-storage contribution in (**a**) NSLPC-800, (**b**) NSLPC-900 and (**c**) NSLPC-1000 at a specific scan rate of 1.2 mV s^{-1}. Contribution ratios of the capacitive process in (**d**) NSLPC-800, (**e**) NSLPC-900 and (**f**) NSLPC-1000 at different scan rates

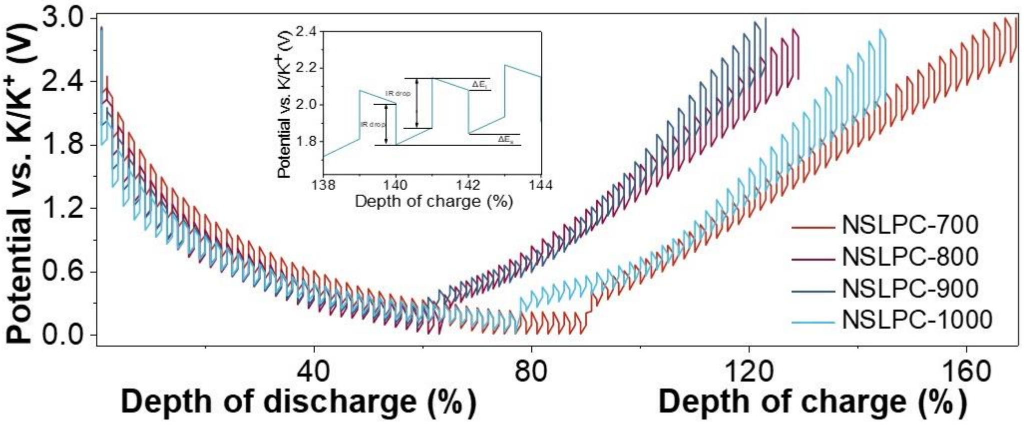

Fig. S29 GITT profiles of the state of the potassiation and depotassiation processes. Testing conditions: this test is tested under the condition of charging or discharging pulses for 5 min each at a constant (pulsed) current density of 0.1 A g^{-1} for 2 hours

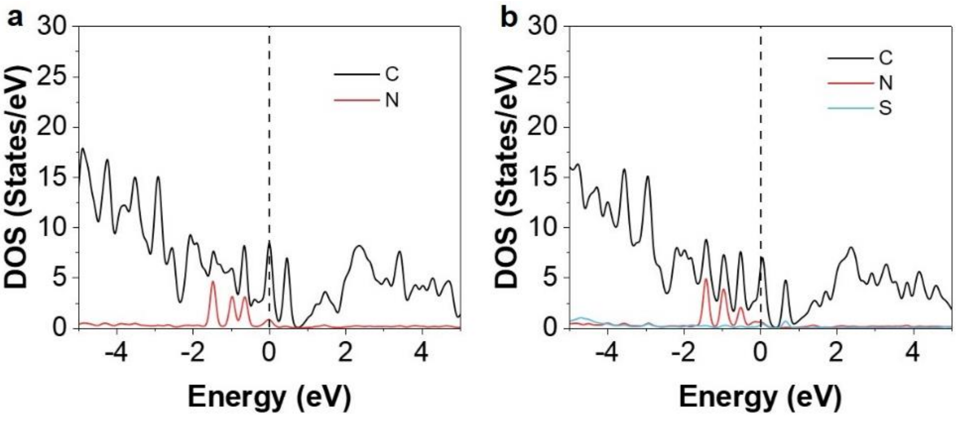

Fig. S30 (**a**) DOS of C and N element. (**b**) DOS of C, N, and S elements

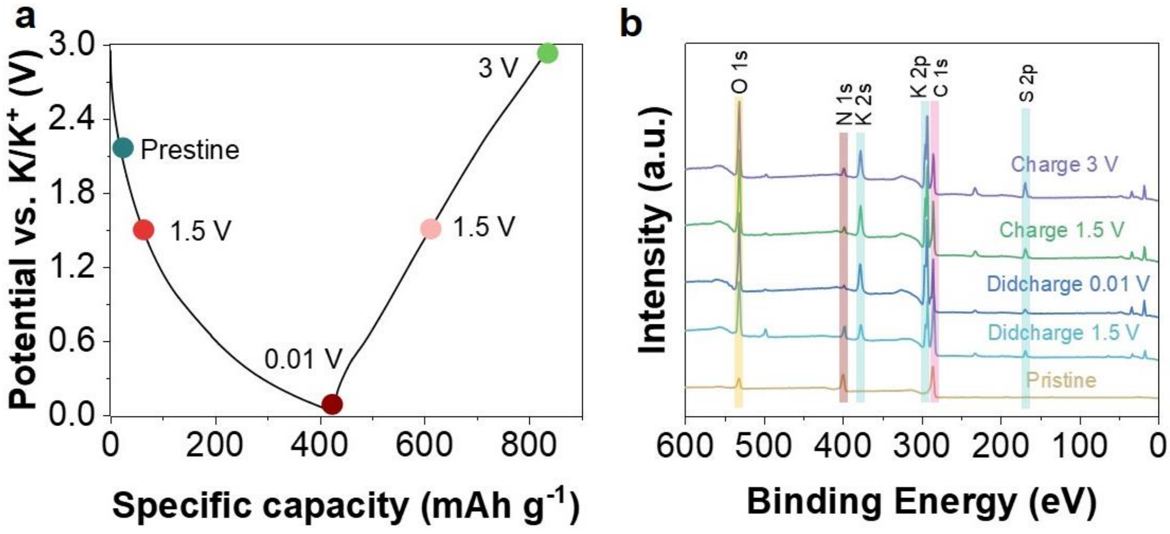

Fig. S31 (**a**) GCD curves of NSLPC-700 at different charge and discharge stages. (**b**) XPS spectra NSLPC-700 at different charge and discharge states

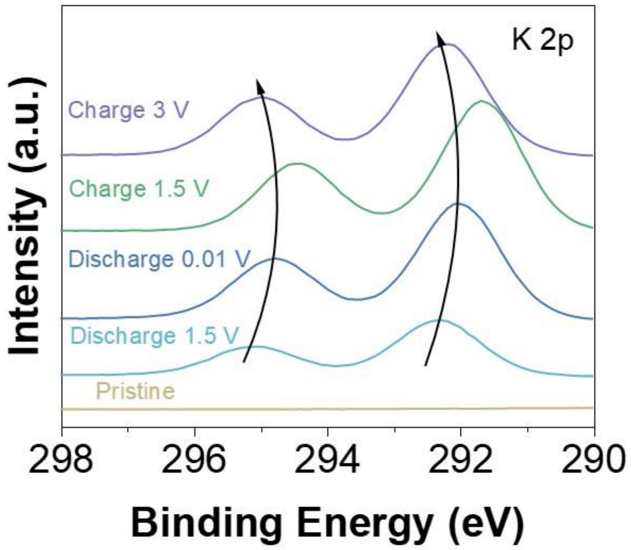

Fig. S32 High-resolution C 1s XPS spectra of NSLPC-700 during the initial discharge and charge cycle

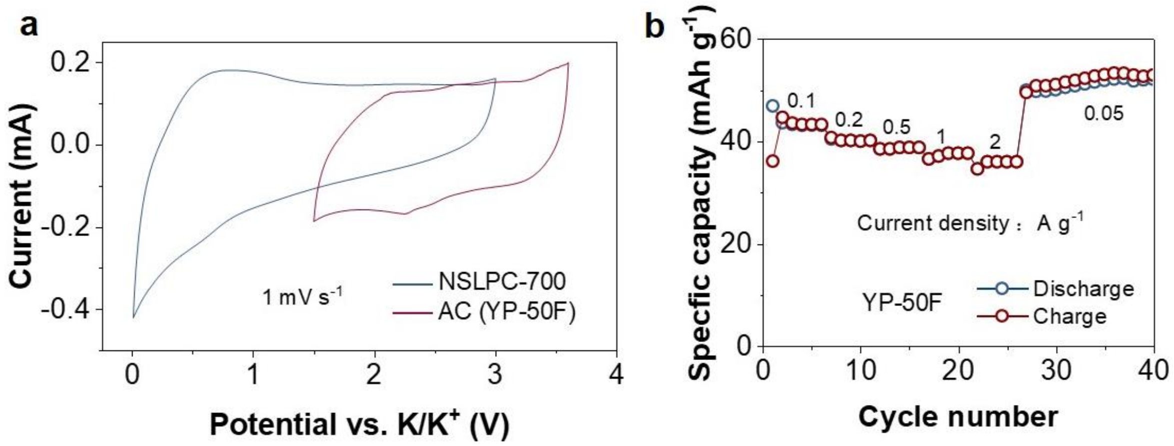

Fig. S33 (**a**) CV curves of the NSLPC-700 and the AC (YP-50F) electrodes in half cells. (**b**) Rate performance of YP-50F cathode

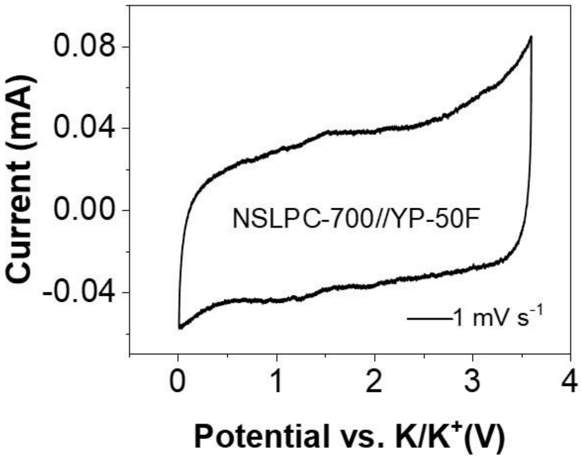

Fig. S34 CV curves of PIHCs assembled by NSLPC-700//YP-50F at a scan rate of 1 mV s^{-1}

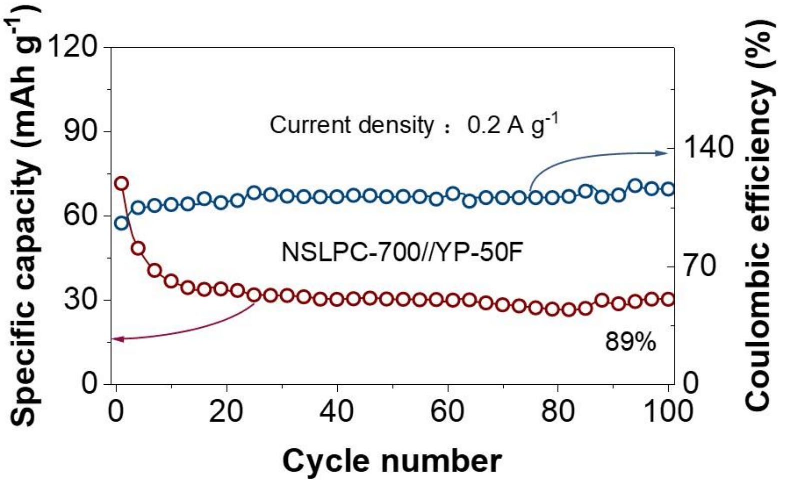

Fig. S35 The cycling performance of PIHCs at a current density of 0.2 A g^{-1} (The specific capacity was calculated based on the total mass of the anode and cathode electrodes)

Supplementary References

[S1] J. Ruan, F. Mo, Z. Chen, M. Liu, S. Zheng et al., Rational construction of nitrogen-doped hierarchical dual-carbon for advanced potassium-ion hybrid capacitors. Adv. Energy Mater. **10**(15), 1904045 (2020). https://doi.org/10.1002/aenm.201904045

[S2] X. Zhang, W. Jian, L. Zhao, F. Wen, J. Chen et al., Direct carbonization of sodium lignosulfonate through self-template strategies for the synthesis of porous carbons toward supercapacitor applications. Colloids Surf. Physicochem. Eng. Aspects **636**, 128191 (2022). https://doi.org/10.1016/j.colsurfa.2021.128191

[S3] X. Chang, X. Zhou, X. Ou, C.S. Lee, J. Zhou et al., Ultrahigh nitrogen doping of carbon nanosheets for high capacity and long cycling potassium ion storage. Adv. Energy Mater. **9**(47), 1902672 (2019). https://doi.org/10.1002/aenm.201902672

[S4] W. Zhang, M. Sun, J. Yin, W. Wang, G. Huang et al., Rational design of carbon anodes by catalytic pyrolysis of graphitic carbon nitride for efficient storage of Na and K mobile ions. Nano Energy **87**, 106184 (2021). https://doi.org/10.1016/j.nanoen.2021.106184

[S5] T. Lu, Molclus. (Beijing Kein Research Center for Natural Science, 2016). http://www.keinsci.com/research/molclus.html

[S6] C. Bannwarth, S. Ehlert, S. Grimme, GFT$_2$-XTB-an accurate and broadly parametrized

self-consistent tight-binding quantum chemical method with multipole electrostatics and density-dependent dispersion contributions. J. Chem. Theory Comput. **15**(3), 1652-1671 (2019). https://doi.org/10.1021/acs.jctc.8b01176

[S7] T. Lu, Q. Chen, Independent gradient model based on hirshfeld partition: a new method for visual study of interactions in chemical systems. J. Comput. Chem. **43**(8), 539-555 (2022). https://doi.org/10.1002/jcc.26812

[S8] T. Lu, F. Chen, Multiwfn: a multifunctional wavefunction analyzer. J. Comput. Chem. **33**(5), 580-592 (2012). https://doi.org/10.1002/jcc.22885

[S9] W. Humphrey, A. Dalke, K. Schulten, VMD: visual molecular dynamics. J. Mol. Graphics **14**, 33-38 (1996). https://doi.org/10.1016/0263-7855(96)00018-5

[S10] G. Kresse, D. Joubert, From ultrasoft pseudopotentials to the projector augmented-wave method. Phys. Rev. B **59**, 1758-1775 (1999). https://doi.org/10.1103/PhysRevB.59.1758

[S11] J.P. Perdew, K. Burke, M. Ernzerhof, Generalized gradient approximation made simple. Phys. Rev. Lett. **77**(18), 3865-3868 (1996). https://doi.org/10.1103/PhysRevLett.77.3865

[S12] S. Grimme, J. Antony, S. Ehrlich, H. Krieg, A consistent and accurate ab initio parametrization of density functional dispersion correction (DFT-D) for the 94 elements H-Pu. J. Chem. Phys. **132**(15), 154104 (2010). https://doi.org/10.1063/1.3382344
